# Understanding the failure process of sulfide-based all-solid-state lithium batteries via operando nuclear magnetic resonance spectroscopy

**DOI:** 10.1038/s41467-023-35920-7

**Published:** 2023-01-17

**Authors:** Ziteng Liang, Yuxuan Xiang, Kangjun Wang, Jianping Zhu, Yanting Jin, Hongchun Wang, Bizhu Zheng, Zirong Chen, Mingming Tao, Xiangsi Liu, Yuqi Wu, Riqiang Fu, Chunsheng Wang, Martin Winter, Yong Yang

**Affiliations:** 1 State Key Laboratory for Physical Chemistry of Solid Surfaces, Collaborative Innovation Center of Chemistry for Energy Materials and Department of Chemistry, College of Chemistry and Chemical Engineering, Xiamen University, Xiamen, 361005 China; 2 School of Engineering, Westlake University, Hangzhou, Zhejiang, 310030 China; 3 College of Energy, Xiamen University, Xiamen, 361005 China; 4 National High Magnetic Field Laboratory, Tallahassee, FL USA; 5 Department of Chemical and Biomolecular Engineering, University of Maryland, College Park, MD 20740 USA; 6 MEET Battery Research Center, Institute of Physical Chemistry, University of Münster, 48149 Münster, Germany; 7 Helmholtz Institute Münster (IEK-12), Forschungszentrum Jülich GmbH, 48149 Münster, Germany

**Keywords:** Batteries, Materials for energy and catalysis, NMR spectroscopy, Electrochemistry, Energy storage

## Abstract

The performance of all-solid-state lithium metal batteries (SSLMBs) is affected by the presence of electrochemically inactive (i.e., electronically and/or ionically disconnected) lithium metal and solid electrolyte interphase (SEI), which are jointly termed inactive lithium. However, the differentiation and quantification of inactive lithium during cycling are challenging, and their lack limits the fundamental understanding of SSLMBs failure mechanisms. To shed some light on these crucial aspects, here, we propose operando nuclear magnetic resonance (NMR) spectroscopy measurements for real-time quantification and evolution-tracking of inactive lithium formed in SSLMBs. In particular, we examine four different sulfide-based solid electrolytes, namely, Li_10_GeP_2_S_12_, Li_9.54_Si_1.74_P_1.44_S_11.7_Cl_0.3_, Li_6_PS_5_Cl and Li_7_P_3_S_11_. We found that the chemistry of the solid electrolyte influences the activity of lithium. Furthermore, we demonstrate that electronically disconnected lithium metal is mainly found in the interior of solid electrolytes, and ionically disconnected lithium metal is found at the negative electrode surface. Moreover, by monitoring the Li NMR signal during cell calendar ageing, we prove the faster corrosion rate of mossy/dendritic lithium than flat/homogeneous lithium in SSLMBs.

## Abstract (web summary)

All-solid-state lithium batteries performance is affected by the solid electrolyte interphase (SEI) and electrically disconnected (“dead”) Li metal. Here, via operando NMR measurements, the authors quantify the Li metal in the SEI and “dead” regions using various inorganic solid-state electrolytes.

## Introduction

All-solid-state batteries (SSBs) hold the promise of achieving high energy density while maintaining safety through the combination of solid state electrolytes (SSEs), high nickel(Ni)-content layered oxide, and lithium metal anode^1–4^. Capacity balancing (negative: positive ratio) is important for the performance and safety of rechargeable battery chemistries^5^. For all-solid-state lithium metal batteries (SSLMBs), a lithium metal thickness of approximately 20 μm is an important prerequisite for realizing SSLMBs with high energy density^6–8^. However, state-of-the-art research widely adopted excess lithium metal anodes (>200 μm), which would lower the energy density of SSLMBs and result in overrated reversibility of lithium metal anodes with oversized capacity at the expense of higher anode weight and volume and thus cell energy density and specific energy. Constraining the use of lithium metal is extensively discussed for lithium metal batteries (LMBs) with a non-aqueous liquid electrolyte solution^9,10^, but rarely mentioned in SSLMBs^8^. There is no doubt that it is more realistic to study SSLMBs with a capacity balanced lithium metal anode, thus, any excess Li is limited or even avoided. The extreme case of a capacity-limited lithium system is the anode-free (“zero-excess”) batteries (AFBs) that consist of a lithium-containing cathode and a bare Cu current collector^11–13^, where the lithium metal anode is formed/deposited in situ during charging. By eliminating the use of expensive and moisture-sensitive thin lithium metal in the discharged state, AFBs are easier to assemble and potentially offer the utmost energy density and specific energy in a cost-effective way^14,15^.

However, the use of a capacity-limited lithium anode degrades its reversibility. Because there is a finite Li source, any formation of inactive lithium will lead to fast capacity decay. The other issue is the formation of high surface area lithium (HSAL) with various Li deposition morphologies^16^, such as “spalling” or “dendritic” lithium, which may penetrate through SSEs to the positive electrode, thus creating a safety-deteriorating short circuit. Researchers have developed various strategies to characterize^17–21^ and suppress^22–25^ the formation of HSAL and inactive lithium, so as to prolong the cycle life of SSLMBs. Despite these efforts, most studies used excess lithium metal and overestimated the reversibility of lithium metal anodes, and the extent of irreversibility remains unknown^26^.

In general, the irreversibility of the lithium metal anode is attributed to the formation of inactive lithium, which comprises of two parts: (i) inactive metallic lithium (also called “dead Li”, i.e., lithium metal regions that are electronically and/or ionically disconnected) that is formed by uneven stripping of lithium metal; (ii) inactive Li^+^ containing compounds generated by the strong interfacial reactivity between lithium metal and the SSEs, very often forming a solid electrolyte interphase (SEI) with immobilized Li^+^, hereafter called “SEI-Li”^27^. Numerous studies were inclined to correlate the composition of SEI with the cycle performance of SSBs^28,29^. Of note, the amount of dead Li and SEI-Li is directly correlated with cell capacity decay, and the current understanding of dead Li vs. SEI-Li in SSBs is limited. Distinguishing and quantifying these two types of inactive Li is the key to understanding the culprits of capacity decay; yet, this task challenges the capabilities of most analytical techniques^30^.

The recently reported titration gas chromatography (TGC) technique offered a postmortem analysis method to quantify inactive lithium that requires battery disassembly^31^. This ex situ method is difficult to extend to SSBs systems, because disassembling SSBs will inevitably damage the interphases and interfaces. Operando nondestructive techniques, including X-ray computed tomography (X-ray CT)^32^, neutron depth profiling (NDP)^33^, nuclear magnetic resonance (NMR) and magnetic resonance imaging (MRI)^34^, have been confirmed to be capable of noninvasively probing the interphase/interior of SSEs. Among them, NMR and MRI are well-suited for this task^35,36^. In particular, operando and in situ NMR spectroscopy coupled with electrochemical testing has been demonstrated as a powerful tool in quantifying inactive lithium in liquid electrolyte LMBs^30,37,38^. Unlike liquid electrolyte LMBs, SSBs require extra stacking pressure to maintain good solid-solid contact. Previous operando NMR/MRI studies for SSBs have widely adopted the Li|SSEs|Li or Li|SSEs|Cu cells^34,39,40^. A more practical cell configuration with lithium transition metal oxide as the cathode has not yet been reported in operando NMR/MRI studies in SSBs. As a result, quantifying inactive lithium has not been achieved in SSBs, and the culprit for capacity decay remains elusive.

Here, we assembled AFBs based on four commonly used ionically conductive and mechanically stable sulfide SSEs^41^, i.e. Li_10_GeP_2_S_12_ (LGPS), Li_9.54_Si_1.74_P_1.44_S_11.7_Cl_0.3_ (LSiPSCl), Li_6_PS_5_Cl (LPSCl) and Li_7_P_3_S_11_ (LPS), to comprehensively investigate the formation of inactive lithium and elucidate their failure mechanisms. The failure mechanisms are systematically analysed and quantified by complementary tools, including operando/ex situ NMR, scanning electron microscopy (SEM), in situ electrochemical impedance spectroscopy (EIS), X-ray photoelectrotron spectroscopy (XPS) and X-ray computed tomography (CT) measurements. The different failure scenarios of each material are proposed. In particular, we demonstrated that all the active lithium converts into SEI-Li in LGPS, while SEI-Li dominates inactive lithium in LSiPSCl, and dead Li is the main culprit in LPSCl and LPS. Two types of dead Li with different formation modes are further identified: one is inside the SSEs due to electrical contact loss, and the other is on the surface of the Cu current collector because of ionic pathway interruption. In addition, we report the Li metal corrosion phenomenon in SSLMBs and demonstrate the faster corrosion rate of dendritic lithium than flat lithium.

## Results

### Electrochemical energy storage performance of all-solid-state anode-free lithium cells

**Fig. 1:**
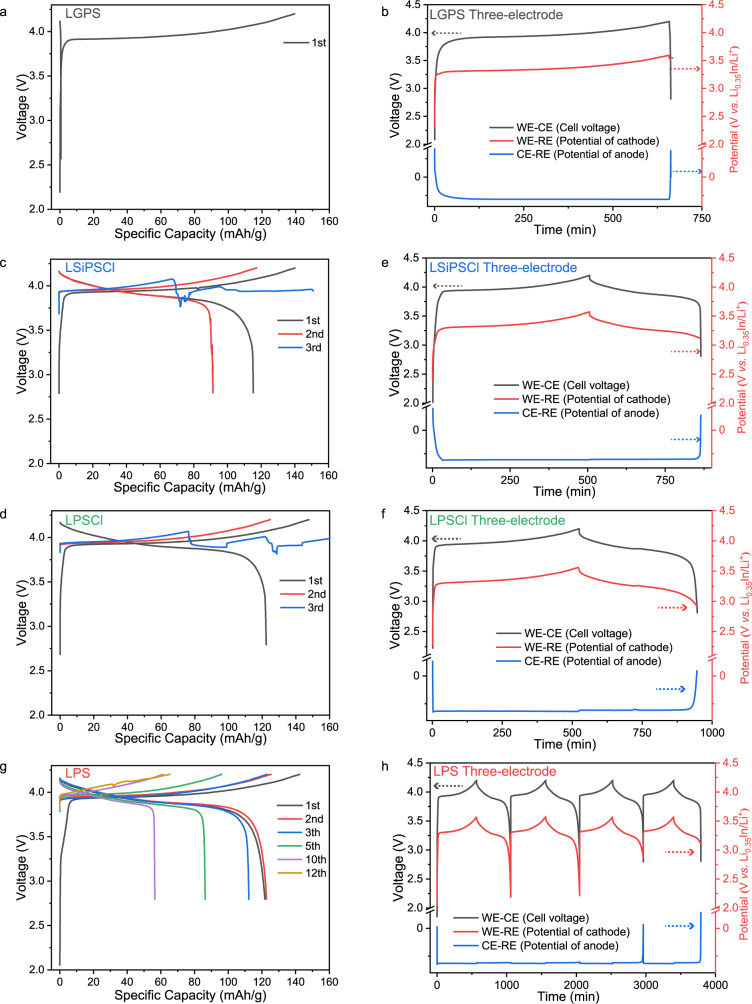
Electrochemical performance of AFBs with four different SSEs. **a** Charge/discharge curves of AFBs with LGPS. **b** Voltage/potential vs. time of the three electrode AFBs with LGPS. **c**, **d** Charge/discharge curves of AFBs with LSiPSCl (**c**) and LPSCl (**d**). **e**, **f** Voltage/potential vs. time of the three electrode AFBs with LSiPSCl (first cycle) (**e**) and LPSCl (first cycle) (**f**). **g** Charge/discharge curves of AFBs with LPS. **h** Voltage/potential vs. time of the three electrode AFBs with LPS (initial four cycles). For three electrode AFBs (homemade mold, similar to Swagelok type), LiCoO_2_ was used as the working electrode (WE), a Cu disc was used as the counter electrode (CE) and Li_0.35_In was used as the reference electrode (RE). The cells are cycled at a current density of 0.08 mA cm^−2^ at 30 ± 2 °C with an applied external pressure of 25 MPa. The mass of “specific capacity” refers to the mass of active material (LiCoO_2_) in the positive electrode.

AFBs (with a “zero-excess-Li” negative electrode) with LiCoO_2_ (LCO as the positive electrode active material) and four types of sulfide SSEs exhibit different electrochemical performance (Fig. 1). The LGPS-based AFBs only show a charging plateau at approximately 3.9 V and cannot deliver any discharging capacity during the first cycle (Fig. 1a). In contrast, the same material tested in a cell with excess lithium metal can cycle multiple times. (Supplementary Fig. 1a). To investigate the cause of the poor AFB electrochemical performance, we tested the electrodes in a three-electrode configuration^42^, in which a lithium indium alloy was used as the reference electrode (see the “Methods” section for details on the three-electrode configuration). The use of a physically different reference electrode helps in differentiating the contribution of anode and cathode potentials to the overall cell voltage^43,44^, as shown in Fig. 1b. The three-electrode cell shows that the potential of the anode rapidly increases at the end of discharging, which causes the overall cell voltage to reach the preset lower cut-off voltage, while the potential of the cathode remains at the charged state. The anode potential rise can be explained by the depletion of active lithium at the anode side, resulting in the absence of active lithium to lithiate the cathode during discharging, thus terminating the cycle life of the LGPS-based AFBs.

LSiPSCl-, LPSCl- and LPS- based AFBs can cycle properly, but short circuits eventually occur and terminate cell operation. Of note, the electrochemical characteristics of these three AFBs are different prior to the short circuit. For LSiPSCl and LPSCl, an abrupt cell voltage drop is seen at the end of dischargeing in the initial two cycles (Fig. 1c, d). Their Coulombic efficiencies are also lower than those of Li-excess cells using the same solid electrolytes (Supplementary Fig. 1 and Supplementary Table 1). The electrochemistry results of their three-electrode cell (Fig. 1e, f) demonstrate that the abrupt voltage drop at the end of the first discharging is due to the quick potential rise of the anode. The high potential of the LCO cathode (vs. RE) at the end of the first discharging (colored in red in Fig. 1e, f) suggests that LCO has not yet fully lithiated, i.e., the lithium ions extracted from LCO during charging cannot be fully inserted back due to the loss of active lithium at the anode side. This differs from the cells using excess lithium metal as the anode, where excess Li serves as a reservoir that ensures that there is enough active lithium to lithiate the cathode, thus, the voltage cut-off is mainly triggered by the change in cathode potential (Supplementary Fig. 2). Therefore, the lower Coulombic efficiencies of AFBs than cells with excess lithium metal are mainly due to active lithium loss on the anode.

For LPS-based AFBs (Fig. 1g and Supplementary Fig. 3), the Coulombic efficiencies of the first two cycles are similar to those of the cell with the excess lithium metal anode, but in the third cycle, it becomes lower than that of the cell using the excess lithium metal anode (Supplementary Fig. 1 and Supplementary Table 1). The three-electrode voltage/potential curves (Fig. 1h) suggest that in the first two cycles, it is the cathode potential drop that triggers the voltage cut-off instead of the anode potential, while from the third cycle onwards, the anode potential rise starts to trigger the voltage cut-off. The electrochemistry data suggest negligible loss of active lithium at the anode side in the first two cycles until the third cycle, where the active lithium loss becomes significant. The abovementioned analysis of electrochemical data indicates that the irreversibility of the reactions occurring at the anode follows the following order: LGPS > LPSCl, LSiPSCl > LPS, with LGPS being the least reversible material.

### Morphological evolution of lithium metal and SSEs during cycling

The morphological evolution of the anode|SSEs interface and the interior of SSEs during cycling was investigated via ex situ scanning electron microscopy (SEM). Supplementary Fig. 4 shows the initial surface and cross-sectional morphology of the four SSEs pellets. The surfaces of LGPS, LPSCl and LSiPSCl are relatively rough and porous, whereas LPS displays a smooth surface with smaller porosity features. Similar morphologies are observed in the cross-sectional view (Supplementary Fig. 4), suggesting that LPS is inherently denser under the same pellet preparation conditions. After the first charging to 4.2 V, the anode was harvested from the cell to examine the morphology of the deposited lithium metal. For LGPS (Supplementary Fig. 5), no lithium metal deposit was observed, neither on the Cu disc surface nor on the surface of SSEs. Instead, there appears to be a non-metallic surface covering the LGPS, which may be associated with the SEI formation.

**Fig. 2:**
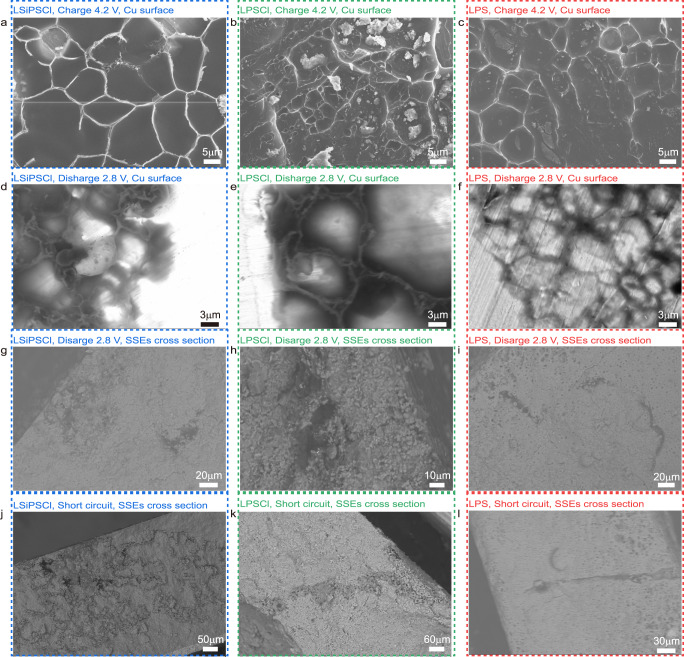
Morphological evolution of the Li metal deposited on the Cu and the cross-section of the representative SSEs. **a**–**c** SEM images of the Cu disc surface after charging to 4.2 V in the first cycle (**a**–**c**). **d**–**l** SEM images of the Cu disc surface (**d**–**f**) and SSEs cross-section (**g**–**l**) using the backscattered electron (BSE) mode, after discharging to 2.8 V in the second cycle (**d**, **e**, **g**, **h**), after discharging to 2.8 V in the third cycle (**f**, **i**) and after the short circuit (**j**–**l**). The Figures (**a**, **d**, **g**, **j**), (**b**, **e**, **h**, **k**), and (**c**, **f**, **i**, **l**) correspond to LSiPSCl, LPSCl, and LPS, respectively.

For the other three sulfide-based AFBs, all deposited Li metal presents a similar mosaic deposit distribution consisting of compact metallic lithium domains, as shown in Fig. 2a–c. After discharging to 2.8 V, interestingly, the mosaic pattern still remains. Lithium metal in the middle section of the mosaic domains is substantially dissolved, with unknown residues remaining in the periphery (Supplementary Fig. 6). For the SEM images obtained in backscattered electron (BSE) mode, these residues exhibit different contrasts in the vertical direction, with the surface appearing brighter and the underneath portions appearing darker (Fig. 2d–f). In BSE mode, lighter elements (e.g., Li in Li metal) present a dark field of view, and relatively heavier elements (e.g., P and S in the SSEs and the SEI) have a brighter contrast. Therefore, the residues on the Cu disc after discharging are tentatively assigned to the unstripped lithium metal (i.e., dead Li) covered with the respective SSEs. In addition to the surface of the Cu disc, some black regions were also observed inside the respective SSEs in the BSE mode, which are supposed to be dead Li inside the electrolyte (Fig. 2g–i). The analysis of the ex situ SEM measurements suggests the presence of two different types of dead Li: residues on the Cu disc and those trapped within the SSEs. After cell short circuit, lithium metal protrusion or the protrusion trajectory (due to the destructive cell disassembly process) towards the cathode are observed in all three systems, as shown in Fig. 2j–l. In particular, a large amount of web-like lithium metal deposition is found in the LSiPSCl system, while extended dendritic lithium metal is observed in the LPS and LPSCl. The AFBs with LSiPSCl after a short circuit were also characterized via ex situ X-ray computed tomography (CT), which clearly revealed the existence of microsized cracks (Supplementary Fig. 7).

### Quantitative analysis of inactive lithium

To gain a quantitative insight into the formation of inactive lithium, we used ^7^Li operando NMR to distinguish and quantify dead Li and SEI-Li formed during the electrochemical cycling of AFBs. Supplementary Fig. 8a shows the ^7^Li NMR spectrum of AFBs (with LPS) after charging to 4.2 V. The chemical shift of lithium metal at approximately 230 ppm is well-separated from the ^7^Li NMR signals of the SSEs and LCO (at approximately 0 ppm). Here, we trace the evolution of the metallic lithium signal to understand the AFBs failure mechanism (Supplementary Fig. 8b). In the initial state, the negative electrode is a bare Cu disc, and no lithium metal signal is seen in the ^7^Li NMR spectrum. After charging to 4.2 V, the increasing signal corresponds to the deposition of Li metal on the Cu disc. After discharging to 2.8 V, the remaining weak lithium metal signal is linked to the irreversible loss of electrochemically active material associated with the formation of dead Li.

**Fig. 3:**
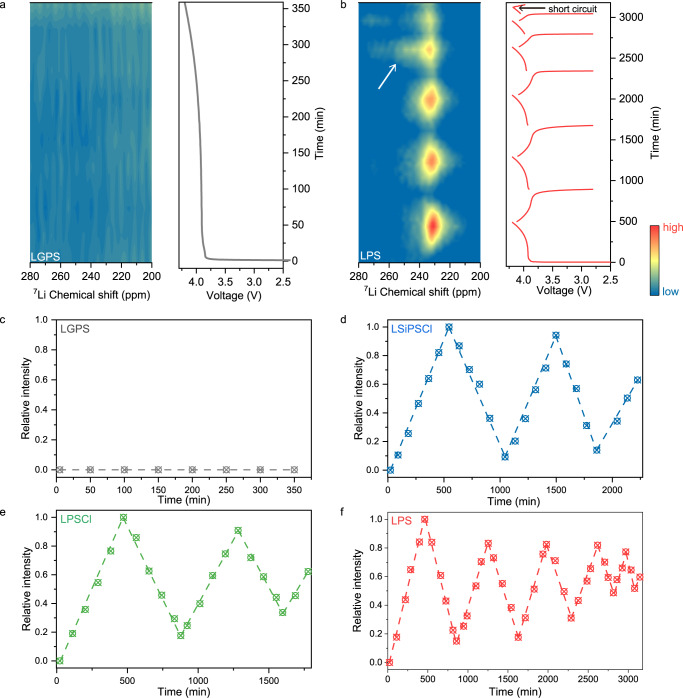
Contour plots of operando ^7^Li NMR spectra and their corresponding charge/discharge curves of AFBs with two different SSEs. **a**, **b** LGPS (**a**), LPS (**b**). The integral of the lithium metal signal versus time, **c**–**f**, for LGPS (**c**), LSiPSCl (**d**), LPSCl (**e**), and LPS (**f**). The operando cells are cycled at a current density of 0.08 mA cm^−2^ at 30 ± 2 °C with an external pressure of 23.6 MPa.

Skin depth (the depth that the radio frequency of NMR can completely penetrate for metallic sample) effect should be evaluated before quantitative analysis of lithium metal signal^37^. The skin depth was previously calculated to be 10.7 μm^30^. The areal capacity of the used cathode is approximately 0.36 mAh cm^−2^, and the thickness of deposited lithium metal was measured to be approximately 3.2 μm via ex situ SEM measurements (Supplementary Fig. 9), which is less than the calculated maximum penetration depth calculated (10.7 μm). Therefore, the quantification based on NMR signal of Li metal is reasonable in our experiments. Figure 3a, b, and Supplementary Fig. 10 show the evolution of the lithium metal NMR signal of AFBs with four SSEs during cycling, and their corresponding integral results are shown in Fig. 3c–f. Surprisingly, for the LGPS (Fig. 3a, c), no lithium metal signal is detected during the entire charging process. For the other three systems, a repeating rise and fall of the lithium metal signal can be observed (Fig. 3b and Supplementary Fig. 10a, b). Then, we compared the integral area of the lithium metal signal at the end of each discharge, which represents the gradual accumulation of the dead Li (Fig. 3d–f). It is apparent that the more accumulation of the dead Li occurs with LPSCl and LPS than with LSiPSCl.

**Fig. 4:**
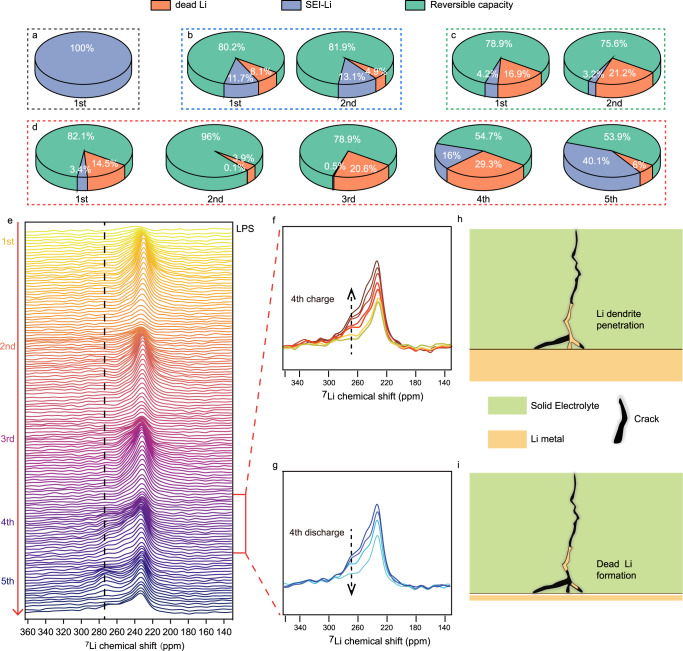
Quantitative and quanlitative analysis of operando ^7^Li NMR spectra of SSBs. **a**–**d** The calculated relative ratio of reversible capacity, dead Li and SEI-Li for cells assembled using LGPS (**a**), LSiPSCl (**b**), LPSCl (**c**), and LPS (**d**). **e**–**g** operando ^7^Li NMR stack spectra of AFB (with LPS) during the first five cycles (**e**), with magnified stacking spectra of the 4th charging process (**f**) and the 4th discharging process (**g**). **h**, **i** Schematic of the lithium dendrite penetration process (**h**) and dead Li formation after subsequent discharging (**i**).

The capacity loss due to dead Li or SEI-Li in the AFBs system can be further differentiated based on the NMR integral area and their electrochemical performance. Basically, electrochemistry data measure the irreversible capacity loss that is related to the total amount of dead Li and SEI-Li being formed. Because dead Li can be quantified by NMR spectroscopy, the amount of SEI-Li can be inferred from the difference between the irreversible capacity loss and dead-Li^30,37^. The calculation process is systematically discussed in Supplementary Note 1, 2 and Supplementary Tables 2–7. The quantitative results of each cycle are summarized in Fig. 4a–d. Because no lithium metal deposit is observed in the LGPS-based AFBs, 100% of the active lithium is considered to be converted to SEI-Li. For LSiPSCl, LPSCl and LPS, both dead Li and SEI-Li contribute to the capacity loss. The contribution of SEI-Li is greater than that of dead Li in each cycle for LSiPSCl, while in LPSCl, dead Li is the main culprit in each cycle. The situation is more complicated for the LPS system, where dead Li mainly contributes to capacity loss in the first three cycles, and then the formation of SEI-Li gradually becomes more pronounced in the fourth and fifth cycles.

The accumulation of dead Li and SEI-Li during cycling is shown in Supplementary Fig. 11a, and the relative ratio of total accumulated dead Li and SEI-Li is shown in Supplementary Fig. 11b. These results also indicate that SEI-Li contributes more to active lithium loss in the case of LSiPSCl (65% for SEI-Li, 35% for dead Li), while dead Li dominates the active lithium loss in LPS (33% for SEI-Li, 67% for dead Li) and LPSCl (17% for SEI-Li, 83% for dead Li). The accumulation of SEI-Li in all four systems is also correlated with an impedance increase. In situ EIS experiments were performed on Li|SSEs|Li batteries during resting (Supplementary Fig. 12, fitting results and fitting errors are shown in Supplementary Fig. 13 and Supplementary Table 8). A cell impedance increase is observed that is mainly due to the formation of the SEI. Thus, a faster impedance increase indicates a less stable SEI^45^. The obtained results demonstrate that deterioration of the Li|LGPS|Li symmetric cell is continuous, whereas in the other three systems, the interface is relatively stable once it is formed (detailed discussion is shown in Supplementary Note 3). The different behaviors are related to the composition of the SEI. The SEI composition of LGPS|Li^18^, LPSCl|Li^20^ and LPS|Li^21^ has previously been studied. Here, we further investigate that SEI composition of LSiPSCl|Li (Supplementary Figs. 14, 15), as discussed in Supplementary Note 3.

In addition to the quantitative information provided by the integral area of the NMR signals, the chemical shift of the Li metal NMR signal is morphologically dependent. The chemical shift of Li metal in different SSEs gradually shifts from ~240 ppm to ~230 ppm during the first cycle charging (Fig. 3b and Supplementary Fig. 10). Of note, such chemical shift is lower than that of in liquid electrolyte-based LMBs (240–275 ppm)^37,46^. We tentatively ascribe the low chemical shift to the synergistic effects of the delithiated cathode and the morphologies of deposited Li (Supplementary Figs. 16, 17 and Supplementary Note 4). Then, we can compare the chemical shift of the deposited lithium metal in different SSEs in the fully charged state, where one can assume that the effect of the delithiated cathode is similar for different SSEs, so that the chemical shift is solely affected by the morphology of deposited Li metal. It can be seen that the chemical shift of deposited Li metal in LSiPSCl is at 227 ppm, while in LPSCl and LPS, it is at 237 and 231 ppm, respectively (Supplementary Fig. 18). It is generally believed that a lower chemical shift represents a flat morphology of Li metal, and a higher chemical shift hints at uneven deposited Li metal or even a dendritic morphology^38,47,48^. These results suggest that lithium metal deposited in the LSiPSCl system has smoother in morphology than lithium metal deposited in the LPSCl and LPS systems.

An interesting phenomenon occurs in the cycle prior to the onset of the short circuit, where part of the metallic ^7^Li NMR signal gradually extends to a higher chemical shift during charging and then gradually decays during discharging, as indicated by white arrows in Fig. 3b and Supplementary Fig. 10. The 1D stacking ^7^Li NMR spectra (with LPS) can be seen in Fig. 4e, and the magnified spectra of the 4th cycle are shown in Fig. 4f, g. A new signal at a high chemical shift spanning from 260 to 280 ppm (indicated by the black dotted arrow) gradually arises during the 4th charge, and its intensity then gradually fades during subsequent discharging, but it does not vanish completely, suggesting the formation of dead Li. A previous report using MRI also observed the appearance of a signal at a high chemical shift, and this signal gradually extended into the interior of SSEs during cycling, which was assigned to the gradual penetration of lithium dendrites^49^. Here, this NMR signal at 270 ppm observed before the short-circuit may be also linked to the growth of lithium dendrites within the SSEs towards the cathode (but not yet in contact with the cathode) during charging (Fig. 4h). In addition, the signal decrease during discharging is thought to be due to the partial dissolution of the dendritic deposit, i.e., dendritic lithium grown into the SSEs cannot be stripped completely, and part of it “breaks off” during the dissolution process and consequently becomes electronically isolated from the current collector, forming dead Li inside the SSEs (Fig. 4i), as also evidenced by the ex situ SEM cross-section images (Fig. 2g–i). Such dynamic evolution process of lithium dendrite formation and dissolution could also be investigated via optical microscopy^16^. This process repeats until a short circuit occurs. Of note, the NMR signal of dead Li consists of more than one signal component, hinting at different types of dead Li metal, which will be discussed in detail in the next section.

### Formation modes of dead Li

**Fig. 5:**
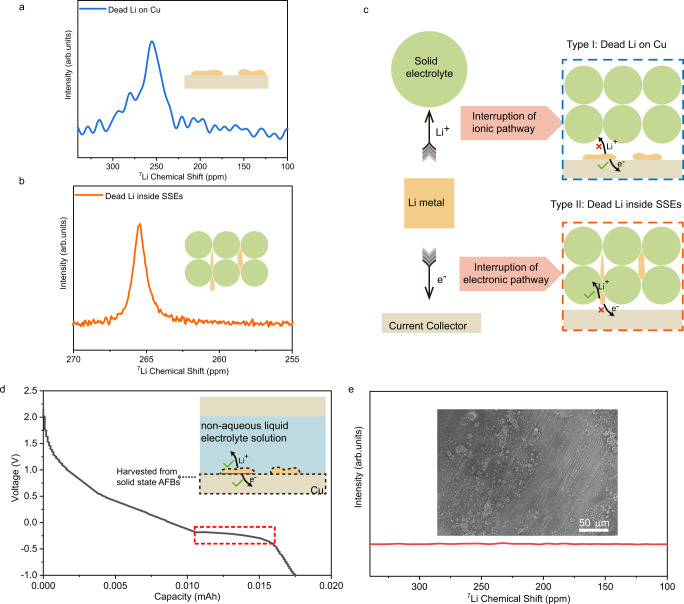
Formation modes of dead Li in the SSBs. **a** ^7^Li NMR spectrum of the recovered Cu disc containing dead Li, performed under static conditions (ex situ, with LPSCl). **b** ^7^Li NMR spectrum of SSEs recovered from cycled AFBs, performed under ex situ MAS conditions, MAS speed: 12 kHz (with LPSCl). **c** Schematic of the two formation modes of dead Li in SSBs: dead Li on Cu and dead Li inside SSEs. **d** Initial voltage curve of a coin cell tested at 0.05 mA cm^−2^ and assembled using a dead Li containing Cu electrode (harvested from an all-solid-state AFBs with LPSCl), a bare Cu counter electrode and a non-aqueous electrolyte solution. The inset is the schematic of the reassembled cell and dissolution of dead Li. After all dead Li is electrochemically dissolved, the voltage further decreases and reaches the lowest cut-off voltage. **e** Ex situ ^7^Li NMR spectrum of the Cu disc retrieved at the end of the discharge process shown in panel (**d**), and its ex situ SEM image is shown in the inset.

The importance of understanding the formation and evolution of dead Li is extensively discussed in the literature^31,50^. However, its existence in SSBs is rarely mentioned due to the use of lithium metal in excess at the negative electrode. In this work, two types of the dead Li are identified via ex situ SEM measurements. The first type is located on Cu, while the other type is found within the SSEs. To further confirm this finding, the Cu disc and SSEs (LPSCl) were harvested separately from a cycled AFBs for subsequent ex situ NMR measurements, in which the Cu disc was sealed in an operando cell for NMR measurement and the SSEs were ground to powder for high-resolution ^7^Li magic angle spinning (MAS) NMR measurement. Both samples present typical NMR signals of lithium metal (Fig. 5a, b), confirming the existence of these two types of the dead Li. Of note, the chemical shift in ex situ NMR cannot be linked with the morphology of dead Li because the disassembly of SSBs would destroy its original morphology.

The deposited Li metal would lose its electrochemical activity because of either electronic or ionic transport pathway interruption. Dead Li inside the SSEs is formed due to physical disconnection from the current collectors, thus losing electronic connectivity and becoming electrochemically inactive (Fig. 5c). A similar formation mode of dead Li has been found in liquid-electrolyte LMBs, where dead Li is wrapped in the SEI and loses contact with the copper current collector, thus interrupting the electronic pathway. However, dead Li is still confined in the non-aqueous electrolyte solution, maintaining the ionic pathway, as illustrated in Supplementary Fig. 19^50^. However, the formation mechanism of dead Li on Cu has not been reported before, and its formation mechanism is still unclear. Based on the ex situ SEM observations (Fig. 2d, f), the dead Li metal attaches tightly to the Cu disc, and it is reasonable to assume that the continuous electronic transport pathways remain intact. Therefore, the reason for the formation of dead Li on Cu is assumed to be the interruption of its ionic transport (Fig. 5c). To verify this hypothesis, we reconnect the ionic transport pathways by pairing a Cu disc containing with dead Li metal that is harvested from solid state AFBs (as a counter electrode) with a fresh Cu disc (as a working electrode) to assemble a Cu-dead Li||fresh-Cu cell using a non-aqueous liquid electrolyte solution (inset in Fig. 5d). This cell can be discharged (Li metal stripping from the Cu-dead Li side), presenting a characteristic plateau at −0.1 V (Fig. 5d), which is comparable with a Li metal stripping process in an asymmetric Li||Cu cells with a non-aqueous liquid electrolyte solution. This experiment demonstrates that “dead Li on Cu” becomes “active” after adding liquid electrolytes. After stripping the dead Li from the Cu disc in liquid electrolyte, the Cu disc was characterized by ex situ NMR and SEM measurements. Lithium metal signal was not observed in the NMR spectrum (Fig. 5e), and the mosaic structure (Fig. 2e) also disappears in the SEM image (inset in Fig. 5e). These experiments demonstrate that the formation of dead Li on Cu is due to the interruption of the continuous ionic transport pathways between Li metal and SSEs in the anode-free SSBs (Fig. 5c). The “dead Li on Cu” (interruption of ionic pathways) exhibits completely different characteristics from that in liquid electrolyte LMBs (interruption of electronic pathways). Moreover, from operando NMR results (Fig. 3c), we can tentatively assign the signal at a chemical shift of 260–280 ppm to dead Li inside the SSEs, and assign the signal at 210–260 ppm to dead Li on the surface of Cu. Therefore, the formation of dead Li on the surface of the Cu disc (interruption of the ionic transport pathway) might be the major source of dead Li in the SSBs.

### Li metal corrosion during calendar aging

**Fig. 6:**
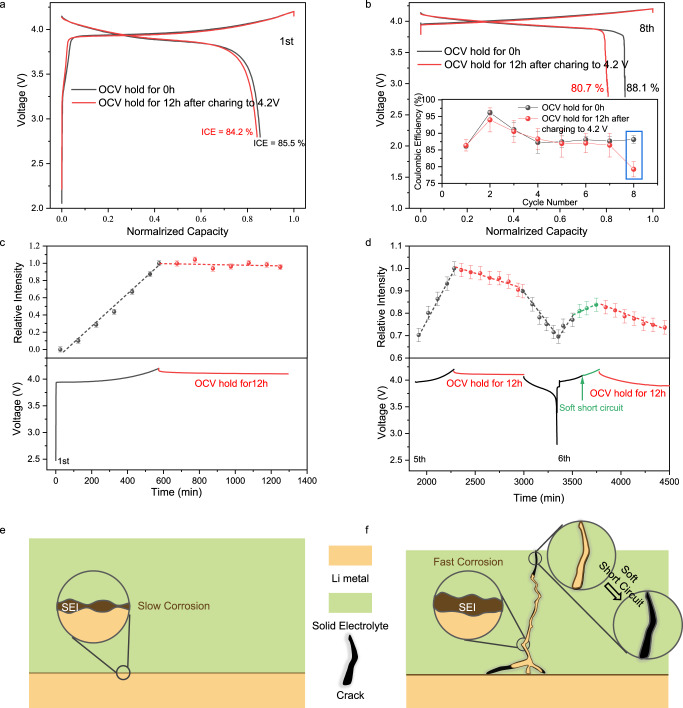
The calendar aging of LPS based AFBs. **a** Charge/discharge curves of the first cycle, with/without 12 h of OCV hold after charging to 4.2 V. **b** Charge/discharge curves of the 8th cycle, with/without 12 h of OCV hold after charging to 4.2 V (inset: evolution of the Coulombic efficiency during cycling). The error bar represents the standard error for three independent experiments. **c**, **d** The integral area of the lithium metal signal versus time and the corresponding charge/discharge curve, with 12 h OCV hold in the first cycle (**c**) and in the 5th and 6th cycles (**d**) after charging to 4.2 V. The error bar represents 30× standard deviation of spectral noise. **e**, **f** Schematic representation of the different corrosion rates of flat lithium (**e**) and dendritic lithium (**f**).

Considering practical applications, calendar aging of the battery, especially for the Li metal anodes, is a key degradation factor that has been investigated in liquid electrolyte based LMBs^37^ but is still neglected in SSLMBs. The LPS-based AFBs were charged to 4.2 V and held at open circuit voltage (OCV) for 12 h at 4.2 V in the first cycle (Fig. 6a), and the same cell OCV holding step is applied for the 8th cycle (Fig. 6b). Comparing cell with and without cell OCV holding step, the initial Coulombic efficiency (ICE) shows little decrease, from 85.5% to 84.2% when cell OCV holding step is used. The Coulombic efficiency of the 8th cycle decreases even more, from 88.1% to 80.7% after cell OCV holding step in the charged state. Such differences suggest different corrosion rates between the first and following cycles.

To quantitatively understand the different Li metal corrosion rates between different cycles, we record the change in the Li metal signal during the cell OCV holding step by operando NMR measurements. For the 1st deposited Li metal, only a single signal at approximately 230 ppm can be seen (Supplementary Fig. 20a), and a slight decrease (approximately 4%) in the lithium metal signal is observed after 12 h of OCV hold (Fig. 6c). In contrast, a new signal, possibly associated with the presence of as dendritic lithium, appears at approximately 270 ppm at the 5th deposition (Supplementary Fig. 20b). 12 h of OCV hold decreases the lithium metal signal by 10% (Fig. 6d), and the main decrease is from the signal of dendritic lithium (Supplementary Fig. 20b). Therefore, the corrosion rates of deposited Li metal are strongly correlated to their morphology, and dendritic lithium has a faster corrosion rate than that of flat lithium. In addition, there is a noticeable voltage fluctuation during the 6th charging process (Supplementary Fig. 21), which is also observed in Fig. 1g and is generally considered a soft short circuit. Although the charging capacity of the 6th cycle is larger than that of the 5th cycle due to the soft short circuit, the lithium metal signal increases slowly after the occurrence of the soft short circuit, and the subsequent discharge capacity is low. These results illustrate that significant active lithium loss occurs after the short circuit, probably because the high current in the cell during the short circuit generates Joule’s heat and “melts” the lithium dendrites, i.e., lithium dendrites lithiate the cathode and/or react with the SSEs under Joule’s heat. This process has also been reported in liquid/polymer electrolyte-based cells^51–53^. The above results show that dendritic lithium is more reactive with LPS than flat lithium, and lithium dendrites can eventually lead to short circuits and result in a large loss of active lithium, as illustrated in Fig. 6e, f. Of note, even in the dendritic case, the corrosion rate of deposited Li metal in SSBs is much slower than that in the previously reported liquid counterpart (10 h of cell OCV hold decreases the amount of deposited Li metal by 25%)^37^, hinting at the advantages of SSBs in protecting Li metal during calendar aging.

## Discussion

Based on the abovementioned qualitative and quantative results, different failure mechanisms can be summarized for the cell chemistries with LGPS, LSiPSCl, LPSCl and LPS. LGPS undergoes the quickest failure. Operando NMR results confirm that lithium metal is not detected during charging for anode-free SSBs. Of note, the observed voltage plateau at 3.9 V during charging is representative of the typical redox couples of LCO delithiation (cathode) and lithium metal deposition (anode). As a result, at the anode side, the lithium metal deposition occurs first and the deposited lithium metal reacts with the LGPS as soon as it is plated, rather than the occurrence of direct reduction of LGPS, as the voltage profile of reduction of LGPS is a slope from 2 V to 0 V^54^. The reaction between deposited lithium and LGPS converts all the active lithium ions provided by the cathode into inactive SEI-Li, which indicates a strong reactivity of the LGPS with lithium metal. Even when the current density is increased to 0.24 and 0.4 mA cm^−2^ to increase the deposition rate of lithium metal, the battery still fails to enable a discharge process (Supplementary Fig. 22), further evidencing the strong chemical reactivity between LGPS and lithium metal. This was also verified by recent ex situ TGC^55^ and operando X-ray CT^29^ (using analogous Li_10_SnP_2_S_12_ in their experiment) techniques. The NMR results in this work complementarily provide quantitative insight into a non-destructive analysis way, and illustrate the adverse effects of using excess lithium metal, which overestimates the electrochemical performance and leads to a misinterpretation of the compatibility and stability of LGPS with lithium metal. The chemical stability between lithium metal and LSiPSCl, LPS and LPSCl is improved compared to LGPS. Such difference is well understood from previous research^56^ that the SEI of LGPS is a mixed conducting interphase due to the existence of Li-Ge alloy, whereas the SEI formed in LSiPSCl, LPSCl and LPS is an ion-conducting but electron-blocking interphase.

The quantitative operando NMR results demonstrate that dead Li contributes more to the active lithium loss in LPSCl and LPS than in LSiPSCl. To understand the factors that influence the formation of dead Li, we summarize the SEI composition, the chemical shifts of deposited lithium (i.e., hinting at the lithium metal morphology) and the relative ratio of total accumulated dead Li and SEI-Li (Supplementary Fig. 11b) of the four systems (Table 1). It seems that the flatter is the morphology of deposited lithium metal, the less dead Li there is, and the morphology of metallic lithium is probably influenced by the composition of the SEI. We believe that the Si-containing SEI in LSiPSCl facilitates the formation of flatter and smoother lithium metal, while the SEI components such as Li_2_S and Li_3_P are linked with uneven lithium deposition. A recent study also introduced Li_x_SiS_y_ as a protection layer on lithium metal that could avoid lithium dendrites and battery short circuits, which supports our viewpoint^57^. Nevertheless, the effect of the SEI composition on the morphology of deposited lithium metal deserves further exploration regarding the mechanism.

**Table 1:** Summary of SEI compositions, chemical shifts of deposited lithium and the relative ratio of total accumulated SEI-Li/dead Li, in four different SSEs

SSEs	SEI composition	SEI-Li ratio	Chemical shift of deposited Li	Dead Li ratio
LGPS	Li_2_S, Li_3_P, Li-Ge alloy^18^	100%	n/a	0%
LSiPSCl	Li_2_S, Si_2_S_6_^6-^, P_2_S_6_^4-^	65%	227 ppm	35%
LPS	Li_2_S, Li_3_P^21^	33%	231 ppm	67%
LPSCl	Li_2_S, Li_3_P, LiCl^20^	17%	237 ppm	83%

Of note, we disclose two types of dead Li:. dead Li on the Cu electrode and dead Li isolated in SSEs. Their formation is likely related to the mechanical properties of the SSEs, i.e., the rigidity and fragility nature of the SSEs. The first type of dead Li formed on the surface of the Cu electrode is isolated from the SSEs. Its formation is likely due to the gradual loss of ionic contact between metallic lithium and SSEs during lithium dissolution or formation of a SEI with low ionic conductivity. Previous studies have also highlighted the issue of electrode|electrolyte contact loss at the interface^58–60^, and they emphasized that the increase of interfacial impedance is due to this contact loss. Here we consider that contact loss can also facilitate the formation of dead Li. The second type of dead Li trapped in the SSEs can be caused by the cracking of SSEs. Lithium dendrites extend into the interior of SSEs through the formed cracks^32,61^ but cannot be completely stripped off due to lack of electronic contact, thus being trapped within SSEs. Therefore, in this case, the easier the SSEs fractures, the more dead Li is likely to be formed. Overall, both the morphology of deposited lithium and the mechanical properties of SSEs have an impact on the formation of dead Li.

In addition to irreversible Li consumption, short circuiting is another severe problem that could lead to the failure of LSiPSCl-, LPSCl-, and LPS-based AFBs. The charging curves show that LPSCl and LSiPSCl were short-circuited in the third cycle, while LPS was short-circuited in the 12th cycle (Fig. 1). The NMR signal of lithium dendrites appears in the second cycle of LPSCl and LSiPSCl, while this signal can be seen in the fourth cycle of LPS (Fig. 3b and Supplementary Fig. 10). These results demonstrate that LPS is more resistant to lithium dendrites than LPSCl and LSiPSCl, which is tentatively attributed to its higher density and lower surface defects. Supplementary Table 9 summarizes several mechanical properties of the SSEs used. We find that LPS has a higher density than LPSCl and LSiPSCl, and the hardness of LPS is slightly higher than that of LPSCl. It is assumed that these properties ensure that LPS has a denser microstructure (Supplementary Fig. 4) and make it more tolerant to lithium dendrites than LPSCl and LSiPSCl. In addition, the surface defects not only induce the formation of lithium dendrites^62^, but also produce crack-tip stresses to promote dendrite penetration^63^. The surfaces of LPSCl and LSiPSCl are relatively rough and porous, while LPS displays a smooth surface with limited porosity, as shown in Supplementary Fig. 4. We conclude that the high density and low surface defects of LPS explain the better performance of LPS than LPSCl and LSiPSCl in AFBs. Battery short circuits always pose serious safety hazards, while in our operando NMR results, a peculiar phenomenon (Fig. 3b, c) was observed before the short circuit happened, which advocates the great potential of the NMR technique in predicting short circuit in SSBs.

The calendar aging experiments give us a clear picture that the corrosion rate of lithium metal in SSBs is lower than that in LMBs using non-aqueous liquid electrolyte solutions, and the flat lithium metal has a slower corrosion rate than the dendritic lithium. Benefitting from the immobility of the SSEs and the dense lithium metal deposits in SSBs, deposited flat lithium has only surface contact with SSEs, which is far less contact than that in liquid electrolyte-based LMBs; thus, the chemical corrosion is reduced in SSBs. Moreover, the current collector is isolated from the SSEs after lithium deposition; thus, perheps the galvanic corrosion (electrochemical process in which Li metal is oxidized and the electrolyte on the surface of the Cu current collector is reduced) is alleviated in SSBs compared to liquid electrolyte-based LMBs. Compared to flat deposited lithium metal, dendritic lithium with a high specific surface area reacts more with SSEs during cell OCV holding step, accelarating its corrosion rate. This also explains why the contribution of SEI-Li is gradually greater than that of dead Li in the 4th and 5th cycles of LPS-based AFBs (Fig. 4d), i.e., more SEI is formed by the reaction of the lithium dendrite with the SSEs.

Based on our understanding of the failure process of the four SSEs, the SSLMBs begin to rapidly deteriorate when the SSEs crack and the subsequent lithium dendrites grow. In this case, more dead Li and SEI-Li are generated and eventually the battery short circuits. Therefore, it is vital to inhibit the formation of lithium dendrites and the cracking of SSEs. According to our abovementioned analysis, two strategies can be further simultaneously pursued: one is to control uniform lithium deposition, and the other is to regulate the mechanical properties of the electrolyte so that it can tolerate greater stress without any cracks. To effectively screen improved strategies, some key scientific questions need to be clarified, including but not limited to (1) lithium nucleation and growth mechanisms in SSBs; (2) the relationship between the SEI composition and lithium metal deposition morphology; and (3) the relationship between the composition of SSEs, various mechanical properties of the SSEs and their tolerance to stress. Finally, we suggest that the battery community should consider more capacity balanced or even anode-free lab-scale cell configurations in future studies to accurately evaluate the electrochemical performance of SSBs.

In conclusion, four sulfide-based SSEs are characterized in AFBs, and each exhibits different electrochemistry behaviors. Quantitative analysis by operando NMR spectroscopy reveals the evolution of dead Li formation as well as the amount of Li-containing SEI component. LGPS reacts immediately with new lithium metal deposits, converting all active lithium into SEI-Li. For LSiPSCl, the formation of SEI-Li is more severe than that of dead Li, whereas dead Li is the major source of irreversibility for LPSCl. For LPS, the formation of dead Li dominates the capacity loss in the first three cycles, and then the formation of SEI-Li becomes greater in the subsequent cycle. The dead Li content is influenced by the morphology of lithium metal deposits. Si-containing SEI in LSiPSCl helps to produce smooth and dense lithium metal deposits, whereas the morphology of deposited lithium metal is inhomogeneous and rough in LPSCl and LPS.

We identified two modes of dead Li: one is on the surface of the Cu current collector due to ionic contact loss, and the other is within SSEs due to loss of electronic contact. Their formation is likely linked to the mechanical properties of SSEs. The lithium metal corrosion rate is found to depend on the morphology of lithium deposits, and dendritic lithium shows higher corrosion rates than flat lithium deposits. All these AFBs show worse cyclability than their Li-excess counterparts. The obtained results comfirmed that the current practice of using excess Li may overestimate the cyclability of SSBs, and developing practical SSBs requires additional analysis of capacity-balanced cells.

## Methods

### Inorganic solid-state electrolytes

Li_10_GeP_2_S_12_ (LGPS), Li_6_PS_5_Cl (LPSCl) and Li_7_P_3_S_11_ (LPS) were purchased from Hefei Kejing Material Technology Co., Ltd. Li_9.54_Si_1.74_P_1.44_S_11.7_Cl_0.3_ (LSiPSCl) was provided by Guilin Electrical Equipment Scientific Research Institute Co., Ltd. A total of 100 mg of the SSEs was pressed into a pellet using a hydraulic machine with 37.5 MPa of pressure, and then carbon-coated Al foil (thickness18 μm, diameter 10 mm, purity of >99.9%, Tianjin Aiweixin Chemical Technology Co., Ltd.) was pressed on both sides of the pellet with 412.5 MPa of pressure to measure the ionic conductivity. Then the thickness of SSEs pellet was measured via micrometre calliper, which was used to calculate their density and relative density (porosity). The assembly and measurement process were performed in an Ar-filled glovebox (H_2_O, O_2_ < 0.01 ppm). The ionic conductivities of LGPS, LSiPSCl, LPSCl and LPS at 30 ± 2 °C are 2.74 mS cm^−1^, 2.88 mS cm^−1^, 2.55 mS cm^−1^ and 1.71 mS cm^−1^, respectively (Supplementary Fig. 23, the fitting errors are shown in Supplementary Table 10).

### Assembly of lab-scale cells with and without excess Li (two-electrode)

The cathode composite was first prepared by mixing Li_4_Ti_5_O_12_-coated LiCoO_2_ (LTO@LCO, purity > 99.9%) (Xiamen Tungsten Co., Ltd) with LGPS at a weight ratio of 70:30 in an agate mortar in an Ar-filled glovebox (H_2_O, O_2_ < 0.01 ppm) for 30 min. The average particle size of LTO@LCO is approximately 5 μm, and there is no carbon coating layer on the LTO@LCO. The Cu disc current collector (Shenzhen Kejing Material Technology Co., Ltd, diameter 10 mm, purity >99.7%, thickness 80 μm) was soaked in 50 mL of dilute hydrochloric acid (0.1 M) for 10 min to remove the surface oxide layer, then cleaned it three times with 20 mL of alcohol (Shanghai Aladdin Biochemical Technology Co., Ltd., purity >99.5%) and quickly transferred to a vacuum oven for drying (pressure: ~150 Pa, temperature: 80 °C, time: 5 h). A total of 100 mg SSE powder (LGPS, LSiPSCl, LPSCl and LPS) was cold pressed via hydraulic machine with 37.5 MPa of pressure in a poly(ether ether ketone) cylinder (PEEK) (inner diameter 10 mm, outer diameter 30 mm, height 100 mm). Then 6 mg of the prepared cathode composite (mass loading of active material: 5.35 mg cm^−2^) was homogeneously spread on one side of the pellet and pressed with 412.5 MPa of pressure. The thickness of SSEs pellet is approximately 800 μm. A Cu disc was placed on the other side of the pellet to assemble AFBs and a lithium metal foil (China Energy Lithium Co., Ltd., thickness 370 μm, purity >99.9%) was pressed to assemble the battery with excess Li. A weight of 0.2 tons (25 MPa) was applied to the cell during the operation of the batteries, which was calibrated with a pressure sensor, as shown in Supplementary Fig. 24a.

The operando solid state cell for NMR was assembled as follows: 60 mg of SSEs powder and 3 mg of cathode composite were used to press a pellet with a diameter of 7 mm under a pressure of 412.5 MPa, and then the obtained pellet and Cu disc were assembled into an operando cell for assembly. Digital images of the operando cell in the assembled and disassembled states are shown in Supplementary Fig. 24b, c. The pressure of the operando cell is applied and maintained by fastening the screw with a torque wrench at 0.95 Nm (Supplementary Fig. 24d, e), which corresponds to approximately 907.8 N (23.6 MPa) (Supplementary Note 5). The abovementioned operations were performed in an Ar-filled glovebox. Three cells were tested in a single electrochemical experiment. The mass of “specific capacity” refers to the mass of active material in the positive electrode.

### Assembly of three-electrode lab-scale cells with a reference electrode

Li_0.35_In was first prepared by pressing lithium (China Energy Lithium Co., Ltd., thickness 370 μm, purity >99.9%) and indium (Shanghai Aladdin Biochemical Technology Co., Ltd., purity >99.99%) together in an Ar-filled glovebox (H_2_O, O_2_ < 0.01 ppm). Aluminum foil (Shenzhen Kejing Material Technology Co., Ltd, purity >99.7%, thickness 80 μm) was cut into small strips and was wrapped by prepared Li_0.3_In, which acted as a reference electrode^64^. A total of 60 mg of SSEs powder was pressed into pellet with 0.3 tons of pressure and the reference electrode was inserted into the homemade cylinder PEEK mold (similar to Swagelok type cell) through a narrow gap in the side wall and was placed on surface of the pellet. Then, another 60 mg of SSEs powder was added to the reference electrode, and 37.5 MPa of pressure were applied so that the reference electrode became embedded in the middle of the SSEs. A 6 mg of prepared cathode composite (mass loading of active material: 5.35 mg cm^−2^) was homogeneously spread on one side of the pellet and pressed with 412.5 MPa of pressure. A Cu disc or a lithium metal foil was placed on the other side of the pellet. A weight of 0.2 tons (25 MPa) was applied to the three-electrode cell during the operation of the batteries. The digital image of the assembled three-electrode cell is shown in Supplementary Fig. 25. Three cells were tested in a single electrochemical experiment. The mass of “specific capacity” refers to the mass of active material in the positive electrode.

### Assembly of Li|SSEs|Li cells

100 mg of SSEs was first pressed into a pellet using hydraulic machine with 412.5 MPa of pressure, and then two lithium metal foils (China Energy Lithium Co., Ltd., thickness 370 μm, purity >99.9%) were attached to the either side of the pellet. A mass of 0.1 tons (12.5 MPa) was applied to the symmetric cell during battery operation. Three cells were tested in a single electrochemical experiment.

### Assembly of Li metal cells with non-aqueous liquid electrolyte solution

The Cu-dead Li||fresh-Cu cell was assembled using a 2025 coin cell. Cu-dead Li was recovered from cycled SSBs by peeling off the negative electrode from the SSEs. Celgard was used as the separator. The electrolyte was 1 M non-aqueous liquid electrolyte solution comprising lithium hexafluorophosphate (LiPF_6_) as the salt, ethylene carbonate:ethyl methyl carbonate (EC:EMC) in a 3:7 weight ratio and, 2 wt.% vinylene carbonate (VC) additive (Shenzhen Capchem Technology Co. Ltd.) and 20 μL was used for cell assembly. The water content of the liquid electrolyte was less than 20 ppm. For the assembly of the operando NMR cell with non-aqueous liquid electrolyte, LiFePO_4_ (LFP) (Shenzhen Kejing Material Technology Co., Ltd, purity >99.7%) was used as the positive electrode, glass fiber (Whatman, thickness: 675 μm, porosity: around 85%) was used as the separator, and a Cu current collector was used as the anode. The cathode slurry was prepared by mixing LFP, polyvinylidene difluoride and carbon black with a weight ratio of 8:1:1 in N-methyl2-pyrrolidone. Then the slurry was coated on Al foil and dried at 80 °C for 5 h in vacuum oven. The mass loading of LFP is 11.8 mg cm^−2^, and the thickness of LFP is around 50 μm. The electrolyte was the same as that of the coin cell. Battery assembling was performed in an Ar-filled glove box with water and oxygen contents below 0.01 ppm.

### Electrochemical measurements

Galvanostatic charging and discharging testing of AFBs, three-electrode lab-scale cells, cells with excess lithium metal and operando NMR solid state cells were carried out on a LAND CT-2001A (Wuhan, China) and a Neware battery (CT-4008, Shenzhen, China) test system at a current density of 0.08 mA cm^−2^, within 2.8–4.2 V. The Cu-dead Li||fresh-Cu cells with the non-aqueous liquid electrolyte solution was discharged to −1 V at a current density of 0.05 mA cm^−2^. The operando NMR cell with non-aqueous liquid electrolyte was charged to 3.8 V at 0.5 mA cm^−2^. The symmetric cell was cycled with 0.1 mA cm^−2^ and 0.1 mAh cm^−2^. EIS was conducted on a Versa STAT MV Multichannel potentiostat/galvanostat (Princeton Applied Research) with a potentiostatic signal. The amplitude is 10 mV, and the frequency range is 1 MHz to 1 Hz for ionic conductivity measurement. The in situ EIS spectra were collected every 2 h from 1 MHz to 0.01 Hz with an amplitude of 5 mV. The number of data points is 60 for ionic conductivity measurement and 81 for in situ EIS. Prior to the EIS test, all batteries were left in open circuit voltage for 20 mins. All electrochemical tests were performed in a climatic chamber at a temperature of 30 ± 2 °C.

### Physicochemical characterizations

#### Ex situ scanning electron microscopy (SEM) sample preparation and measurements

Ex situ SEM measurements (Hitachi S-4800) were performed to investigate the deposition and dissolution morphologies of lithium metal in different SSEs, and backscattered electron mode (SEM, ZEISS Sigma) was used to analyze dead lithium or lithium dendrites existed on the SSEs surface and in cross-sections. Of note, the disassembly of SSBs would inevitably destroy the morphology of interphase, deposited lithium and SSEs. Therefore, the application of ex situ SEM to solid-state electrolytes and electrodes definitely requires careful sample preparation to obtain a representative morphology. The sample preparation was discussed in Supplementary Note 6 and the digital images after battery disassembly were shown in Supplementary Fig. 26. Using Hitachi S-4800, samples were transferred under argon atmosphere, while using ZEISS Sigma, samples were transferred quickly, i.e., exposed to air for a short period of time (10 s).

#### Nuclear magnetic resonance (NMR) spectroscopy measurements

Operando NMR measurements were carried out with a homemade operando probe and operando cell^65^ using a Bruker Advance III 400 MHz spectrometer. A single pulse sequence was conducted to acquire the 1D spectrum, and the π/2 pulse and recycle delay were 16 μs and 0.8 s, respectively. A total of 17 min is needed to acquire one spectrum and the interval time between every two spectra is 8 min. The chemical shifts of LGPS (0.2 ppm), LSiPSCl (1.1 ppm), LPSCl (0.5 ppm) and LPS (0.2 ppm) are used as references. For the operando experiments of AFBs with non-aqueous liquid electrolyte solutions, the small angle pulse and recycle delay were 6 μs and 1 s, respectively^30^. The operando cell is placed horizontally (*x*-*y* plane) with respect to the external magnetic field (*z*-axis) (Supplementary Fig. 16a), and its orientation is maintained for all experiments. Ex situ NMR was carried out with a 4 mm MAS probe under the spinning rates of 12 kHz. The solid state electrolytes after cycling were grinding into powder and filled into rotor for ex situ NMR test. The ^7^Li MAS NMR spectrum was collected with single pulse sequence, π/2 pulse and recycle delay were 6 μs and 1 s, respectively. For the preparation of Li_0.5_CoO_2_ electrode, the two-electrode solid state anode free cells (LiCoO_2_ as the cathode) was firstly assembled (see “Assembly of lab-scale cells with and without excess Li (two-electrode)”). Then the cell was charged to 4.2 V and stopped, where the cathode corresponds to formula of Li_0.5_CoO_2_. Finally, cathode composite was recovered by scraping the cathode composite from cell with a scalpel.

#### X-ray computed tomography (CT) measurements

Ex situ X-ray CT measurements (Cheetah EVO, YXLON) were used to study internal microcracks in the SSEs. After the battery cycling (for cell assembly details, see the “Assembly of lab-scale cells with and without excess Li” section), the SSEs pellet was kept embedded in the PEEK tube for CT scanning in order to avoid damage to the SSEs pellet from cell disassembly.

#### X-ray photoelectron spectroscopy (XPS) measurements

Ex situ XPS measurements were carried out using Thermo Scientific Escalab 250Xi^+^, which was calibrated to the C 1 s peak at 284.8 eV of the hydrocarbon contamination. The symmetric cell (two lithium electrodes tightly adhered to the SSEs pellet) was taken out of the cell mold after cycling, then peeling off the two lithium electrodes from the symmetric cell with a scalpel to expose the SSEs pellet. The surface of the SSEs pellet was used for XPS measurements. The samples were quickly trasfered into the XPS chamber, i.e. exposed to air for a short period of time (15 s).

### Reporting summary

Further information on research design is available in the Nature Portfolio Reporting Summary linked to this article.

## Supplementary information

Supplementary Information

Reporting Summary

## Data Availability

The data that support the findings of this study are available from the corresponding author upon reasonable request.
