# Supplementary material for: Understanding the failure process of sulfide-based all-solid-state lithium batteries via operando nuclear magnetic resonance spectroscopy
Source: Nat Commun. 2023 Jan 17;14:259. doi: 10.1038/s41467-023-35920-7 (PMC9845218; doi:10.1038/s41467-023-35920-7)
Supplement: Supplementary file 1 — Supplementary Information [file 41467_2023_35920_MOESM1_ESM.pdf]

Supplementary Information

Understanding the failure process of sulfide-based all-solid-state lithium batteries via operando nuclear magnetic resonance spectroscopy

Ziteng Liang[1], Yuxuan Xiang[1,2], Kangjun Wang[1], Jianping Zhu[1], Yanting Jin[1], Hongchun Wang[3], Bizhu Zheng[1], Zirong Chen[1], Mingming Tao[1], Xiangsi Liu[1], Yuqi Wu[3], Riqiang Fu[4], Chunsheng Wang[5], Martin Winter[6,7], Yong Yang[1,3]*

[1]State Key Laboratory for Physical Chemistry of Solid Surfaces, Collaborative Innovation Center of Chemistry for Energy Materials and Department of Chemistry, College of Chemistry and Chemical Engineering, Xiamen University, Xiamen, 361005, China.

[2]School of Engineering, Westlake University, Hangzhou, Zhejiang 310030, China.

[3]College of Energy, Xiamen University, Xiamen, 361005, China.

[4]National High Magnetic Field Laboratory, Tallahassee, FL, USA.

[5]Department of Chemical and Biomolecular Engineering, University of Maryland, College Park, MD 20740, USA.

[6]MEET Battery Research Center, Institute of Physical Chemistry, University of Münster, 48149 Münster, Germany.

[7]Helmholtz Institute Münster (IEK-12), Forschungszentrum Jülich GmbH, 48149 Münster, Germany.

Corresponding author: Yong Yang, email: yyang@xmu.edu.cn

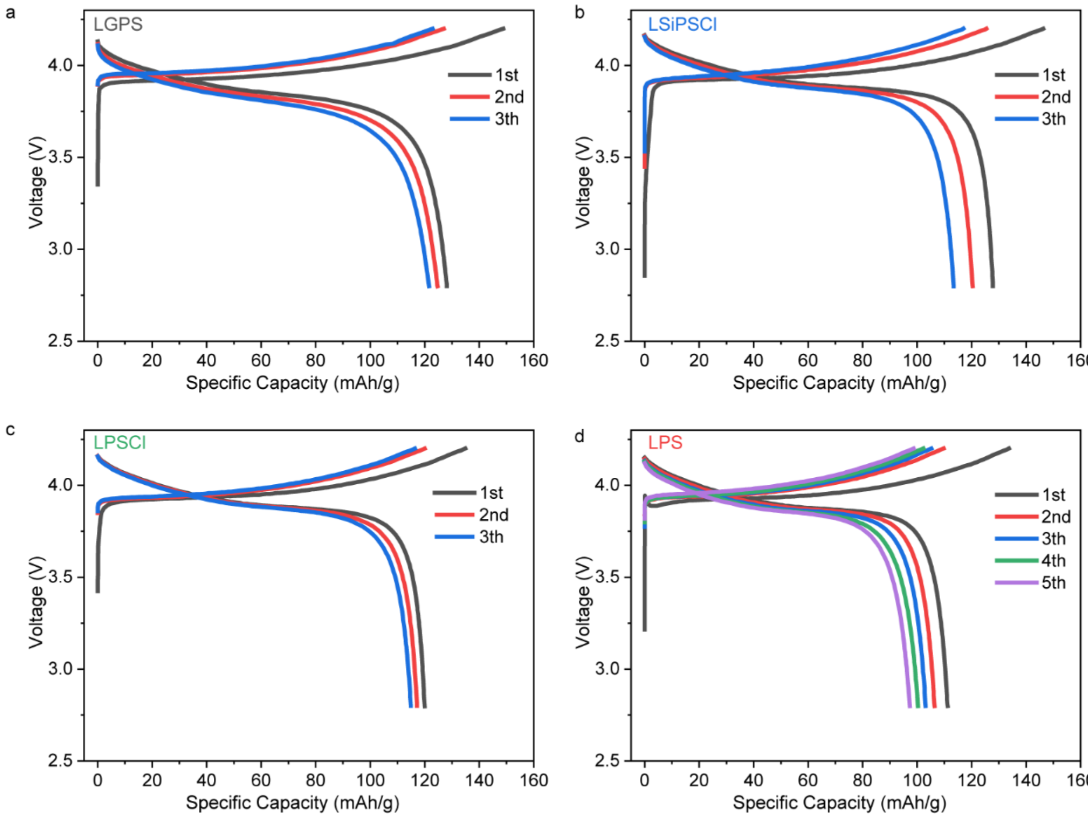

Supplementary Figure 1. a-d, Charge and discharge curves of Li metal cell (with excess Li) using LGPS **(a)**, LSiPSCl **(b)**, LPSCl **(c)**, LPS **(d)** solid electrolyte. The cells are cycled at a current density of 0.08 mA cm^{-2}, at 30 ± 2 °C, with an applied external pressure of 25 MPa. The mass of "specific capacity" refers to the mass of active material ($LiCoO_2$) in the positive electrode.

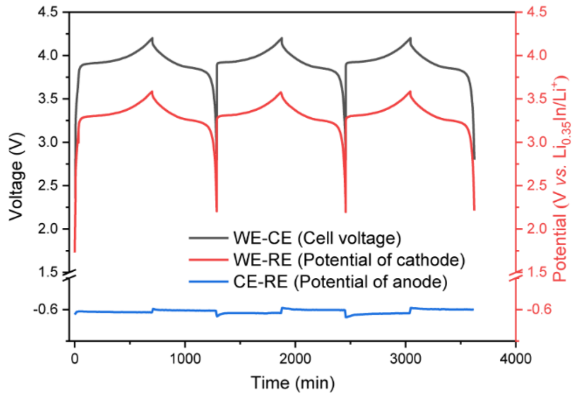

Supplementary Figure 2. Potential *vs.* time of the initial three charge/discharge cycles of the three-electrode cell with excess lithium metal using LSiPSCl. For three electrode cells (homemade mold, similar to Swagelok type), $LiCoO_2$ was used as the working electrode (WE), lithium metal was used as the counter electrode (CE) and $Li_{0.35}In$ was used as the reference electrode (RE). The three-electrode cells are cycled at a current density of 0.08 mA cm^{-2}, at 30 ± 2 °C, with an applied external pressure of 25 MPa.

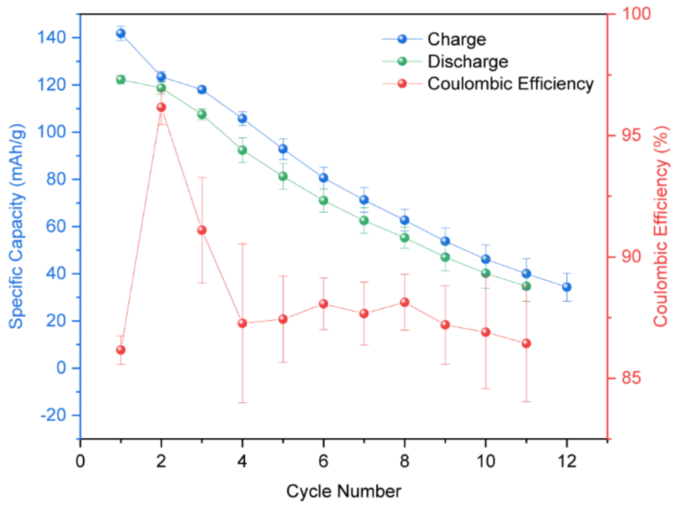

Supplementary Figure 3. Cycling performance of LPS-based AFBs, which are cycled at a current density of 0.08 mA cm^{-2}, at 30 ± 2 °C, with an applied external pressure of 25 MPa. The error bar represents the standard error for three indepedent experiments.

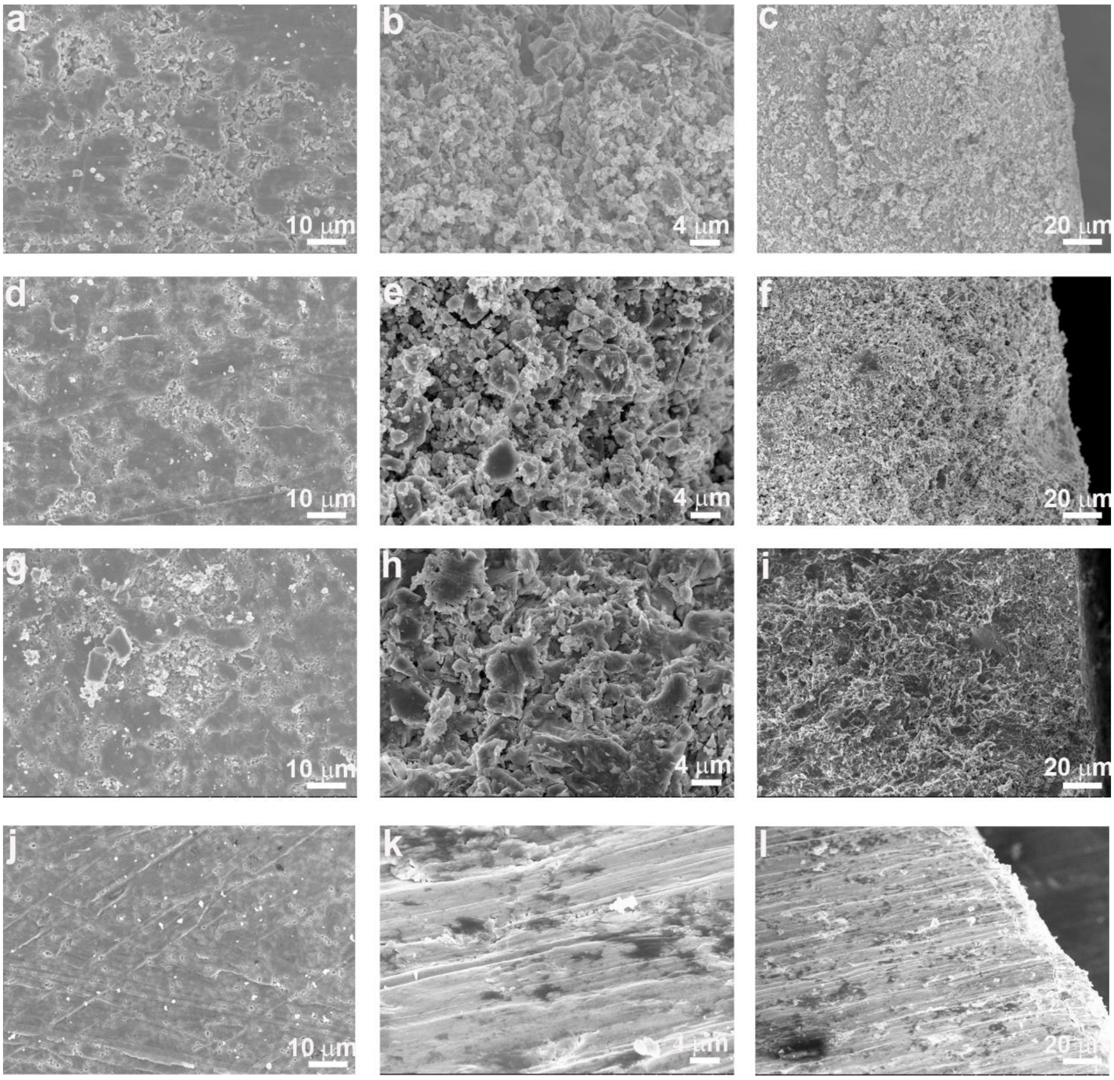

Supplementary Figure 4. Morphologies of surface and cross-sections of SSEs pellets. a-c, SEM images of the LGPS pellet surfaces **(a)** and cross-sections **(b and c)**. **d-f,** LSiPSCl pellet surfaces **(d)** and cross-sections **(e and f)**. **g-i,** LPSCl pellet surfaces **(g)** and cross-sections **(h and i)**. **j-l,** LPS pellet surfaces **(j)** and cross-sections **(k and l)**. The solid electrolyte powder is pressed into pellet with an applied external pressure of 412.5 MPa. The prepared pellets are used for surface morphological characterization. The pellets are broken apart for cross-sections morphological characterization.

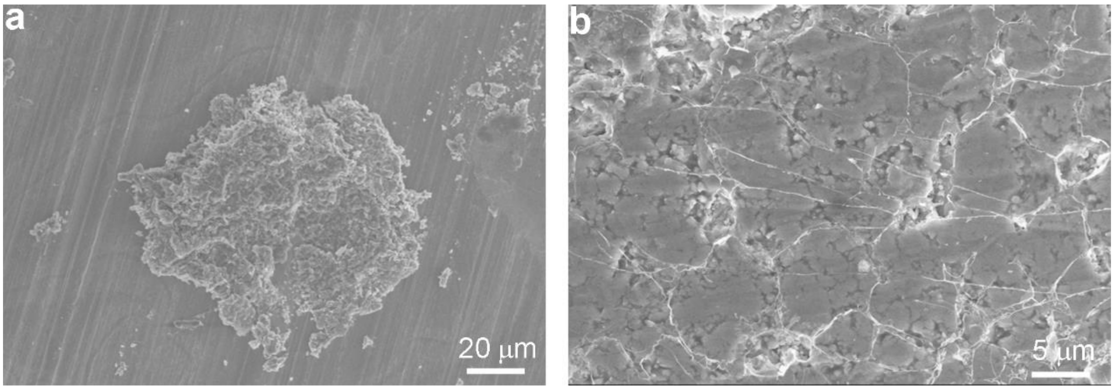

Supplementary Figure 5. SEM images of the copper disk surface **(a)** and LGPS pellet surface **(b)** retrieved from AFBs (using LGPS) after charging to 4.2 V of the first cycle. The cells are cycled at a current density of 0.08 mA cm^{-2}, at 30 ± 2 °C, with an applied external pressure of 25 MPa.

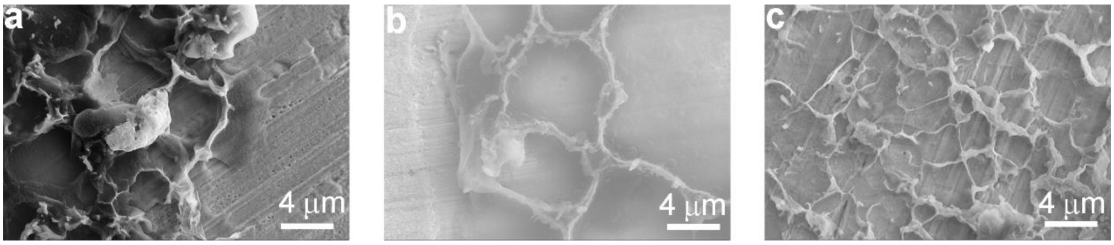

Supplementary Figure 6. SEM images of the copper disk surface of AFB. a-c, after discharging to 2.8 V in the second cycle with LSiPSCl **(a)** and LPSCl **(b)**, after discharging to 2.8 V in the third cycle with LPS **(c)**. The cells are cycled at a current density of 0.08 mA cm^{-2}, at 30 ± 2 °C, with an applied external pressure of 25 MPa.

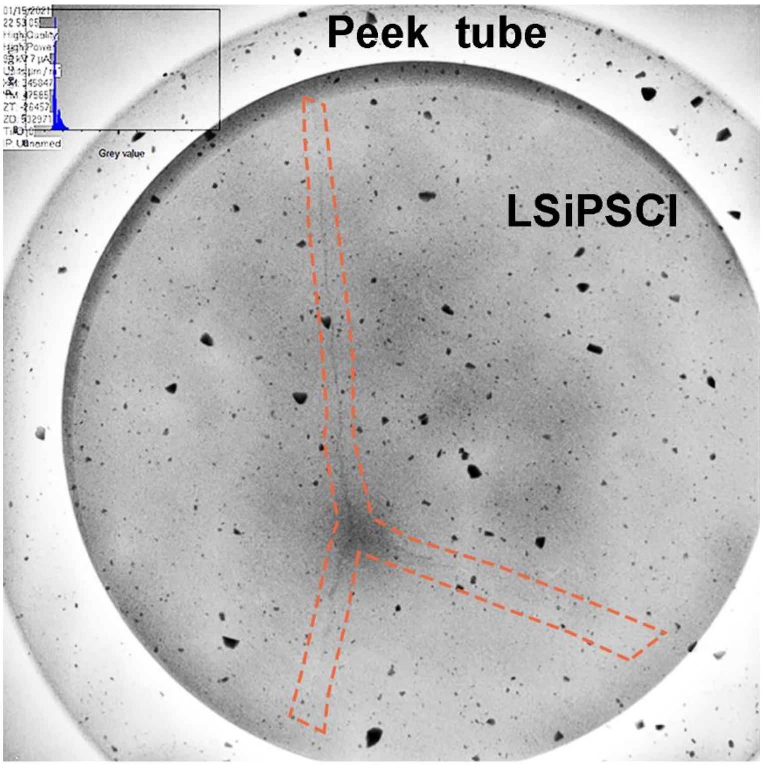

Supplementary Figure 7. X-ray CT of AFBs with LSiPSCl after short circuit. The AFBs are cycled at a current density of 0.08 mA cm^{-2}, at 30 ± 2 °C, with an applied external pressure of 25 MPa. The X-ray CT measurement is carried out after cell short circuit (three cycles). The orange dotted line refers to the formed crack inside SSEs after cell short circuit.

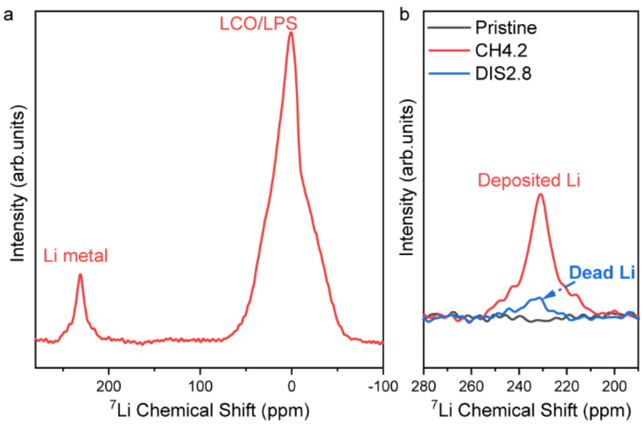

Supplementary Figure 8. (a) Operando ^{7}Li NMR spectrum (with LPS) after charging to 4.2 V. (b) Operando ^{7}Li NMR spectrum (with LPS, only showing the lithium metal signal) in the pristine state, after charging to 4.2 V and after discharging to 2.8 V. The operando cells are cycled at a current density of 0.08 mA cm^{-2}, at 30 ± 2 °C, with an applied external pressure of 23.6 MPa.

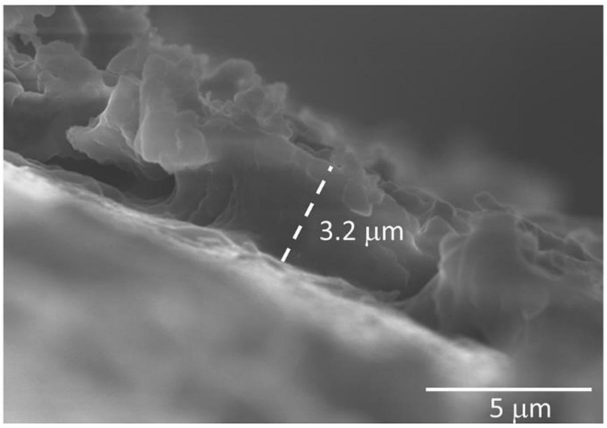

Supplementary Figure 9. SEM image of cross-section of deposited lithium in LSiPSCl-based operando AFBs. The thickness of deposited lithium is around 3.2 μm. The operando cells are cycled at a current density of 0.08 mA cm^{-2}, at 30 ± 2 °C, with an applied external pressure of 23.6 MPa. The negative electrode was harvested after charging to 4.2 V of the first cycle.

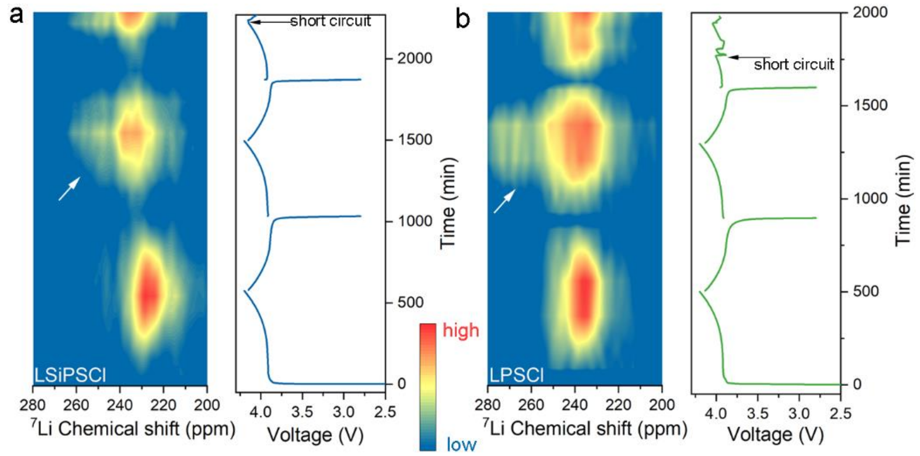

Supplementary Figure 10. Contour plots of operando ^{7}Li NMR spectra and their corresponding charge/discharge curves of AFBs with two kinds of SSEs. a and b, LSiPSCl **(a)**, LPSCl **(b)**. The operando cells are cycled at a current density of 0.08 mA cm^{-2}, at 30 ± 2 °C, with an applied external pressure of 23.6 MPa. The wide arrows indicate the appearance of NMR signal at higher chemical shift.

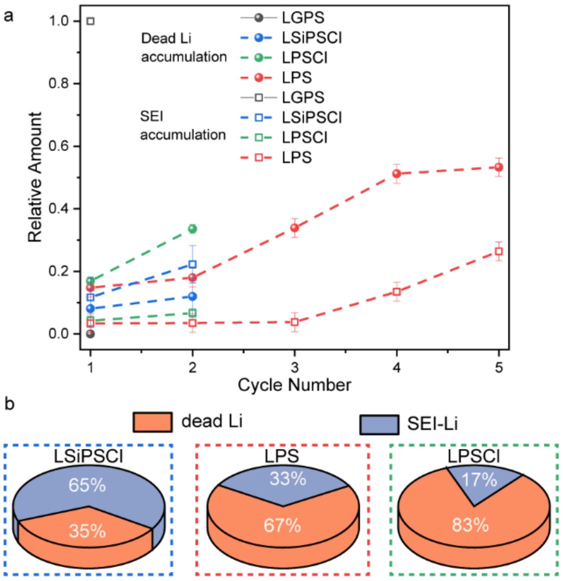

Supplementary Figure 11. a, Accumulation of dead Li and SEI during cycling. The relative amount is calculated as the capacity due to the formation of dead Li or SEI-Li divided by the charge capacity achieved at the first cycle. The error bar represents 30× standard deviation of spectral noise. **b,** The relative ratio of total accumulated dead Li and SEI-Li. The total accumulated dead Li/ SEI-Li is the sum of irreversible capacity from dead Li/SEI-Li in first two cycles for LSiPSCl and LPSCl, and in first five cycles for LPS. The operando cells are cycled at a current density of 0.08 mA cm^{-2}, at 30 ± 2 °C, with an applied external pressure of 23.6 MPa.

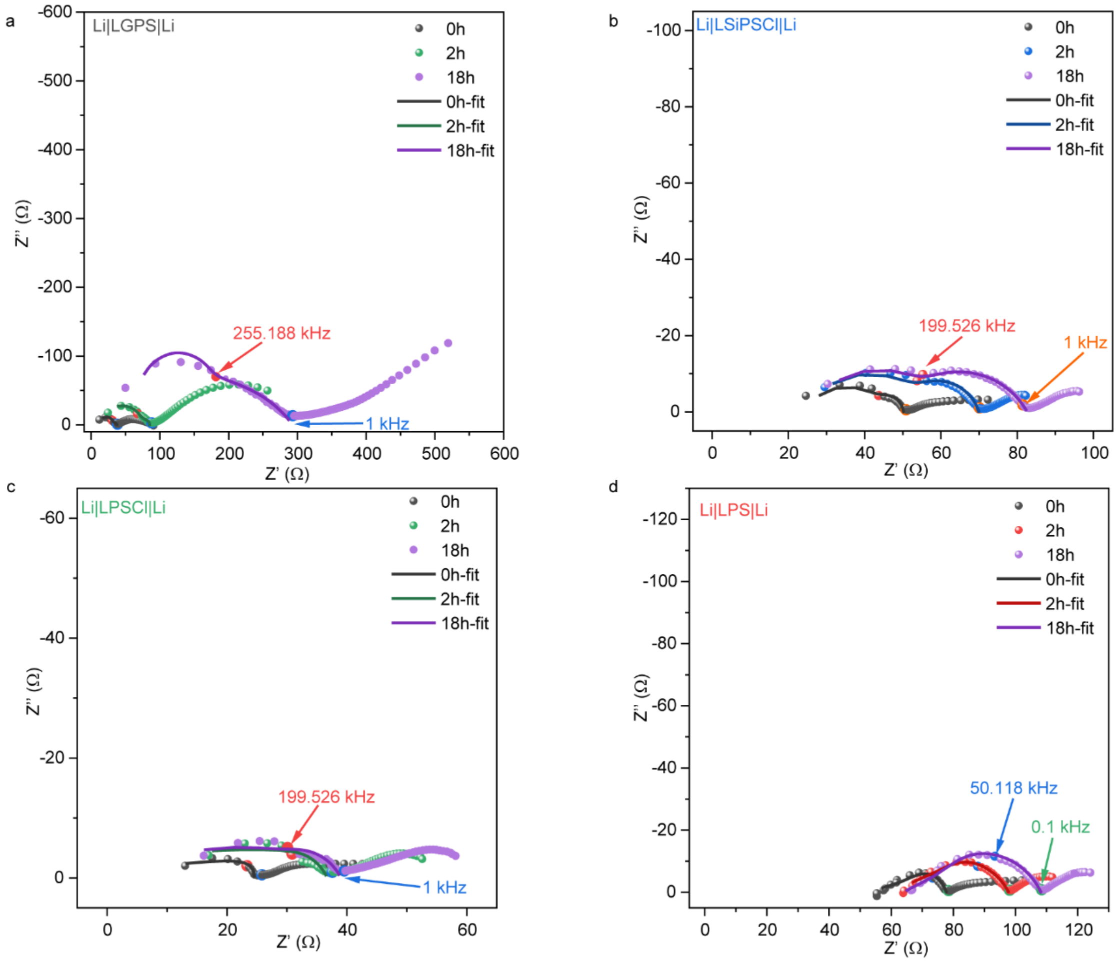

Supplementary Figure 12. In situ EIS spectra of Li|SSEs|Li symmetric cell during resting. a – d, EIS spectra atdifferent rest times of LGPS (a), LSiPSCl (b), LPSCl (c), LPS (d). The error bar represents the error between the raw and fitted data. The symmetric cells are tested at 30 ± 2 °C and with an applied external pressure of 12.5 MPa. Prior to the EIS test, all batteries were left in open circuit voltage for 20 mins.

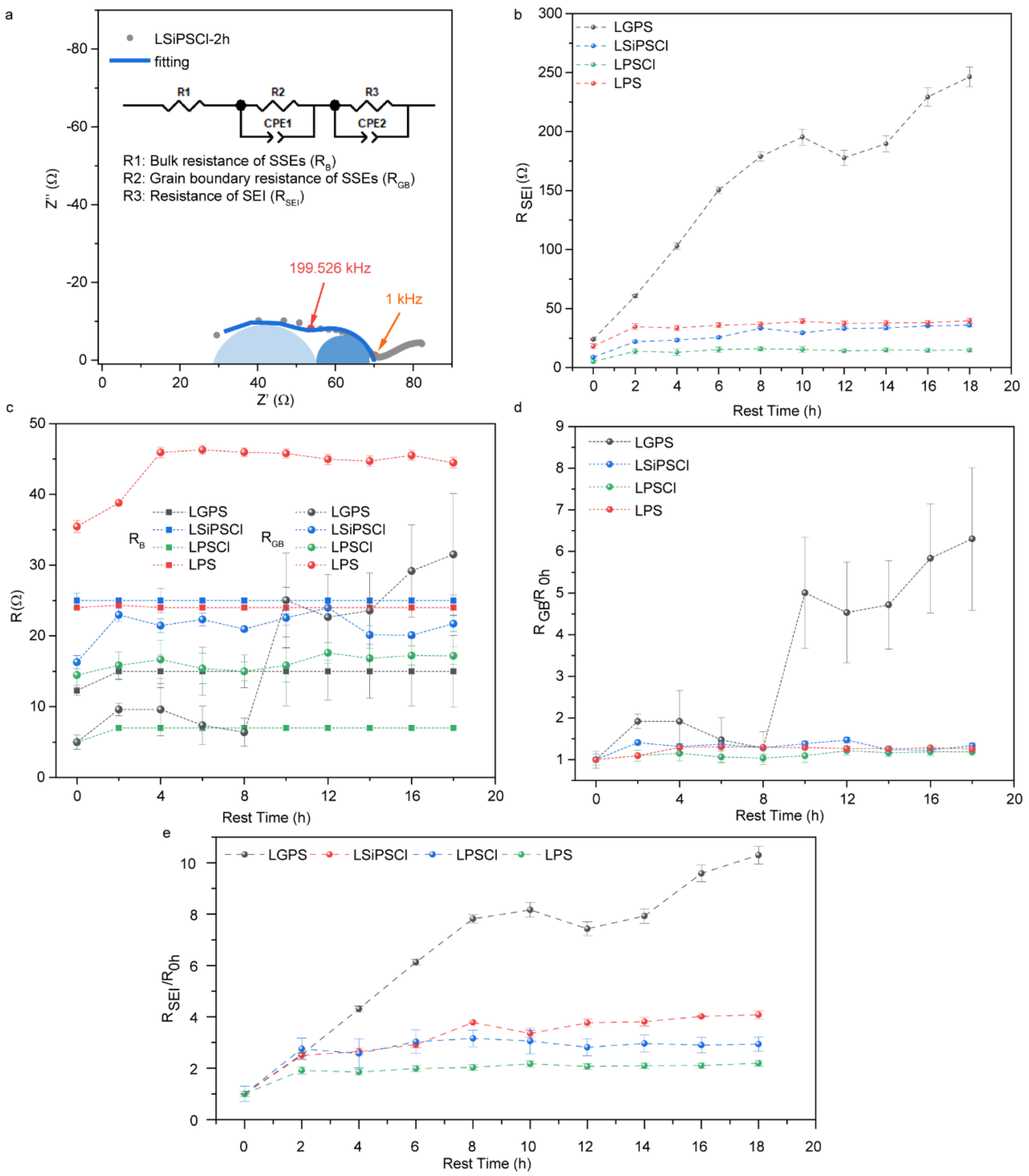

Supplementary Figure 13. a, EIS spectrum and the corresponding fitting results of a Li|LSiPSCl|Li symmetric cell after 2h resting, the inset is the equivalent circuit used for fitting. **b, c,** The evolution of the impedance from SEI formation (R_{SEI}) (b) and bulk resistance (R_B) and grain boundary resistance (R_{GB}) of SSEs (c) during resting period for these four SSEs. **d,** the fitted normalized resistance of grain boundary resistance (R_{GB}) of SSEs of the four systems. **e,** the fitted normalized resistance of SEI formation (R_{SEI}) of the four systems. The error bar represents the error between the raw and fitted data. The symmetric cells are tested at 30 ± 2 °C and with an applied external pressure of 12.5 MPa. Prior to the EIS test, all batteries were left in open circuit voltage for 20 mins.

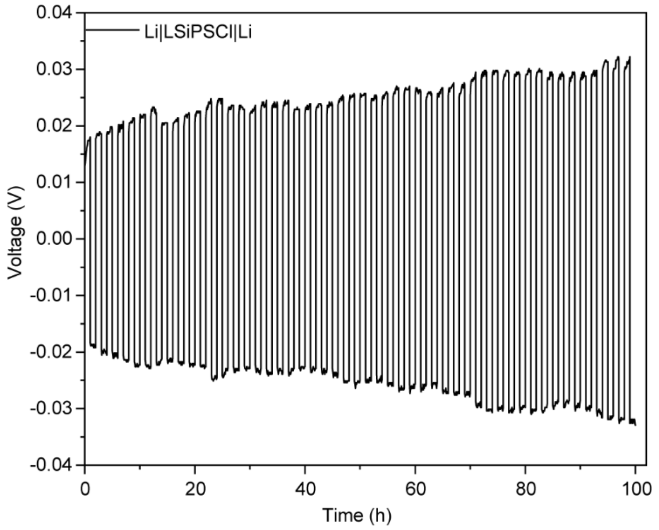

Supplementary Figure 14. Electrochemical performance of a Li|LSiPSCl|Li cell cycling at 0.1 mA cm^{-2}/0.1 mAh cm^{-2} at 30 ± 2 °C, with an applied external pressure of 12.5 MPa.

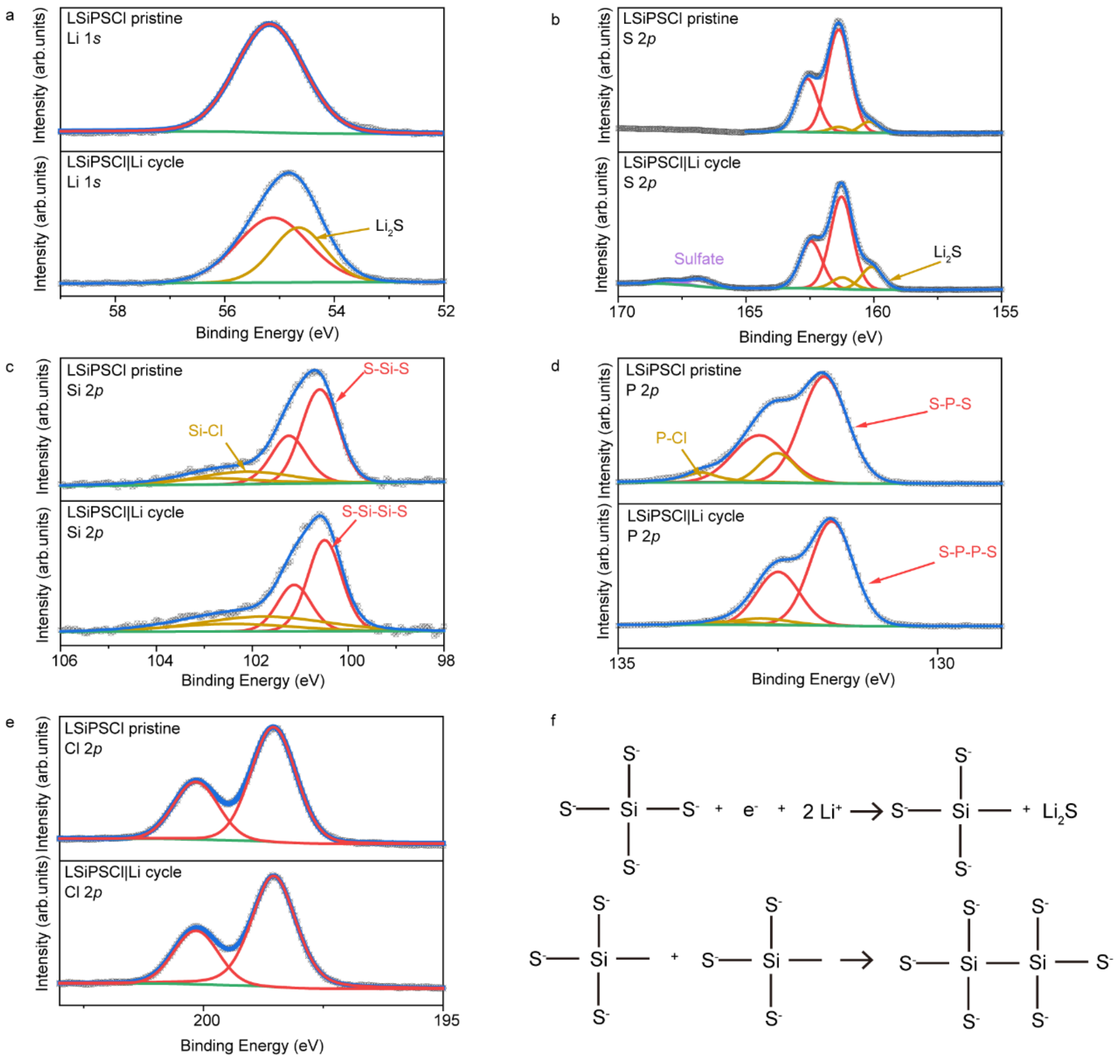

Supplementary Figure 15. The ex situ XPS spectra of Li|LSiPSCl|Li symmetrical cell before and after cycling. a-f, Li 1*s* spectra **(a)**, S 2*p* spectra **(b)**, Si 2*p* spectra **(c)**, P 2*p* spectra **(d)**, Cl 2*p* spectra **(e)**, chemical equation for the reduction of SiS_4^{4-} to $Si_2S_6^{6-}$ **(f)**. Li|LSiPSCl|Li cells were cycled with 0.1 mA cm^{-2} and 0.1 mAh cm^{-2}, at 30 ± 2 °C, with an applied external pressure of 12.5 MPa. The cells were disassembled for XPS measurements after cycling for 100 h.

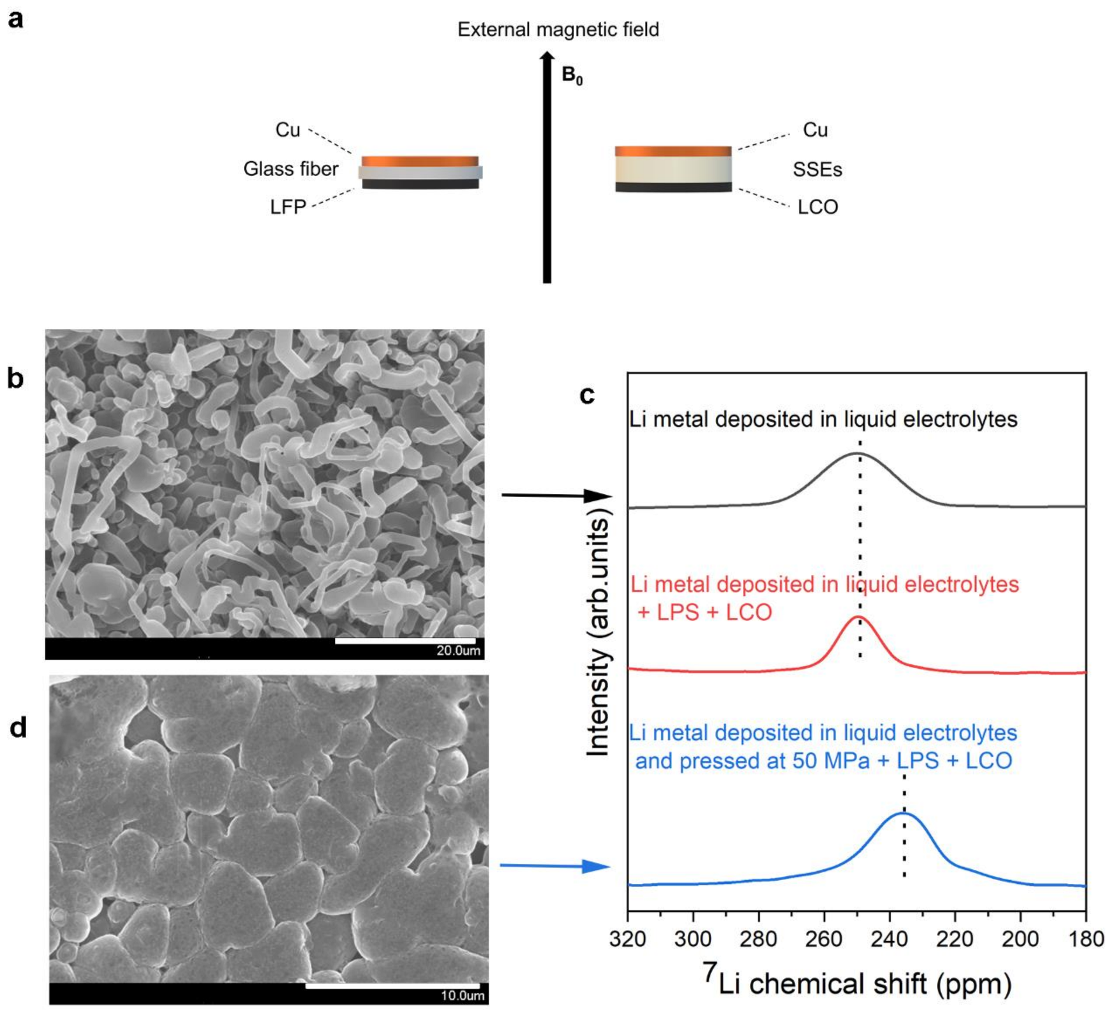

Supplementary Figure 16. a, Schematic of cell orientation with respect to the external magnetic field. **b,** Ex situ SEM measurements for Li metal electrode deposited in an operando NMR cell with non-aqueous liquid electrolyte solution **c,** The ex situ NMR spectra of lithium metal in different experimental conditions. **d,** Ex situ SEM measurements for Li metal electrode that was firstly deposited in an operando NMR cell with non-aqueous liquid electrolyte solution, then harvested and pressed at 50 MPa. LFP refers to $LiFePO_4$.

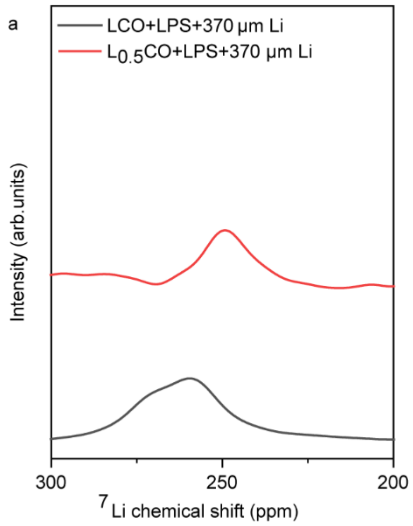

Supplementary Figure 17. a, ex situ ^{7}Li NMR spectra of uncycled Li strip with thickness of 370 μm when pairing with LCO/$L_{0.5}CO$ (partially delithiated $LiCoO_2$, LCO charging to 4.2 V) and LPS. The cells are charging to 4.2 V at a current density of 0.08 mA cm^{-2}, at 30 ± 2 °C, with an applied external pressure of 25 MPa.

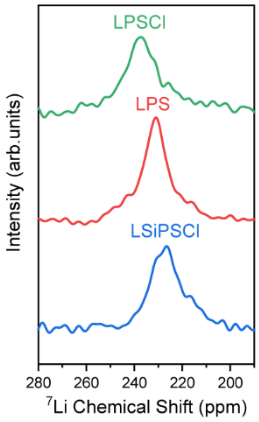

Supplementary Figure 18. Operando ^{7}Li NMR spectra of deposited lithium metal on Cu in AFBs with different SSEs after charging to 4.2 V in the first cycle. The operando cells are cycled at a current density of 0.08 mA cm^{-2}, at 30 ± 2 °C, with an applied external pressure of 23.6 MPa.

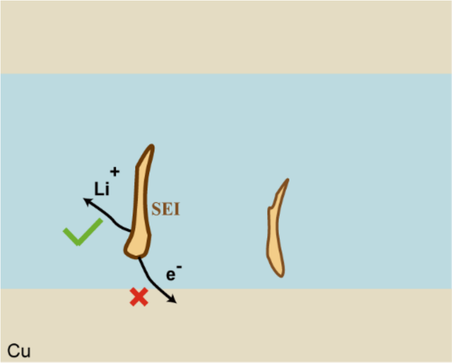

Supplementary Figure 19. Schematic representation of dead Li (i.e., the orange geometrical figures) in a non-aqueous electrolyte solution (i.e., the light blue area) confined within two copper electrodes (i.e., the light grey areas). Dark brown lines represent the SEI formed around the Li metal particles

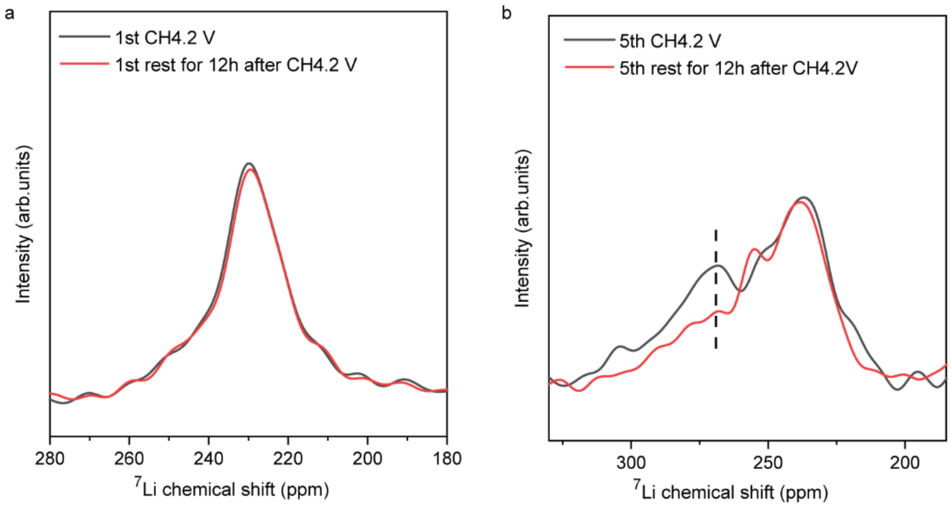

Supplementary Figure 20. Operando ^{7}Li NMR spectra of LPS-based AFBs before and after rest. a, in the first charge and rest process. b, in the 5th charge and rest process. The operando cells are cycled at a current density of 0.08 mA cm^{-2}, at 30 ± 2 °C, with an applied external pressure of 23.6 MPa.

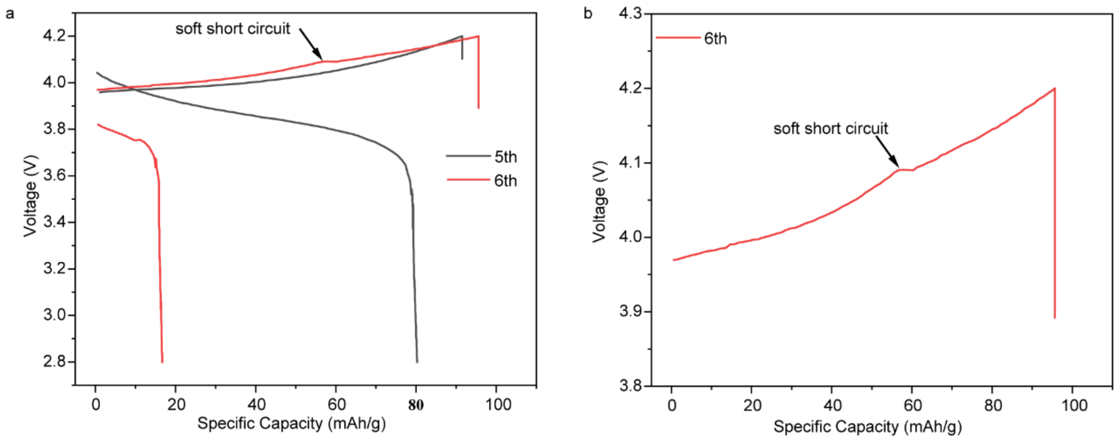

Supplementary Figure 21. Charge and discharge curves of LPS based AFBs (for operando NMR experiments) resting 12h after charging to 4.2 V. a, The 5th and 6th cycle **(a)**, enlarged view of the 6th charge **(b).** The operando cells are cycled at a current density of 0.08 mA cm^{-2}, at 30 ± 2 °C, with an applied external pressure of 23.6 MPa.

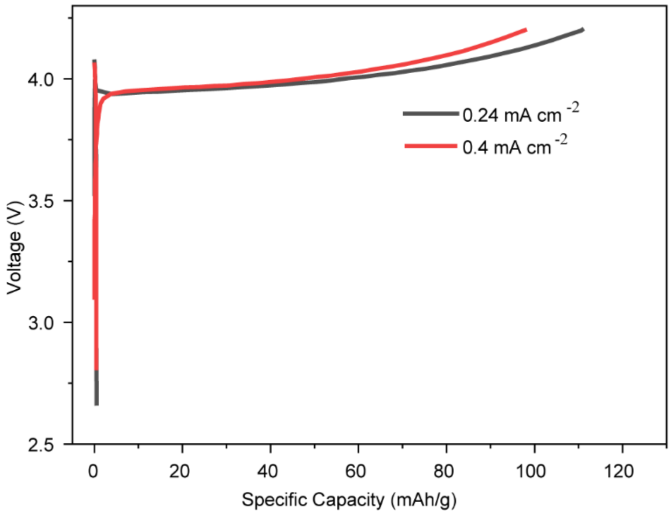

Supplementary Figure 22. Charge/discharge curve of AFBs with LGPS cycled at a current density of 0.24 and 0.4 mA cm^{-2}. The AFBs are cycled at 30 ± 2 °C, with an applied external pressure of 25 MPa.

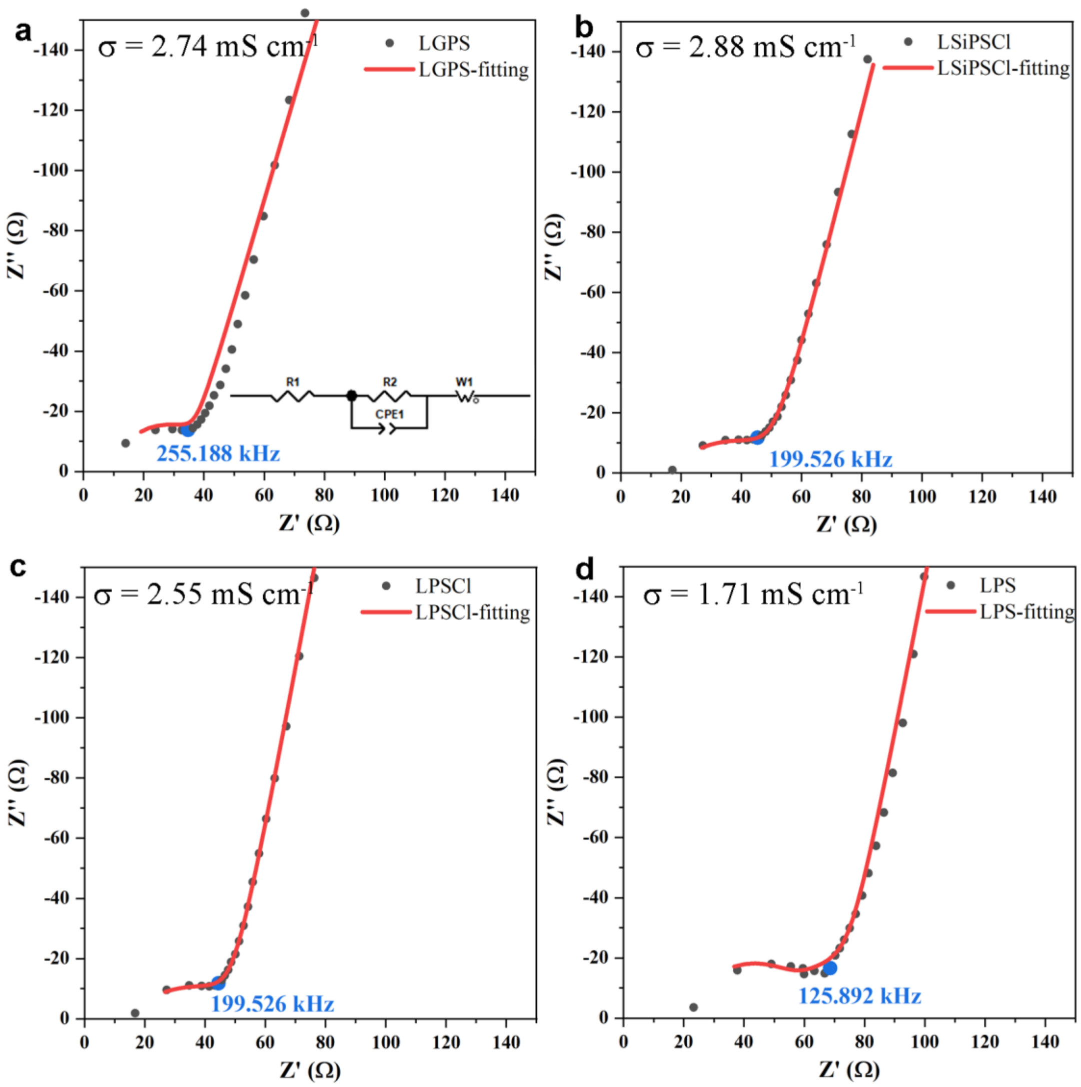

Supplementary Figure 23. a-d, Complex impedance plots and fitting results of C|SSEs|C (C refers to carbon coated Al foil that is served as blocking electrode) battery with LGPS **(a)**, LSiPSCl **(b)**, LPSCl **(c)** and LPS **(d)**. The inset in a) shows the equivalent circuit model for data fitting. The pellet density is summarized in Supplementary Table2. The cells are tested at 30 ± 2 °C, with an applied external pressure of 25 MPa.

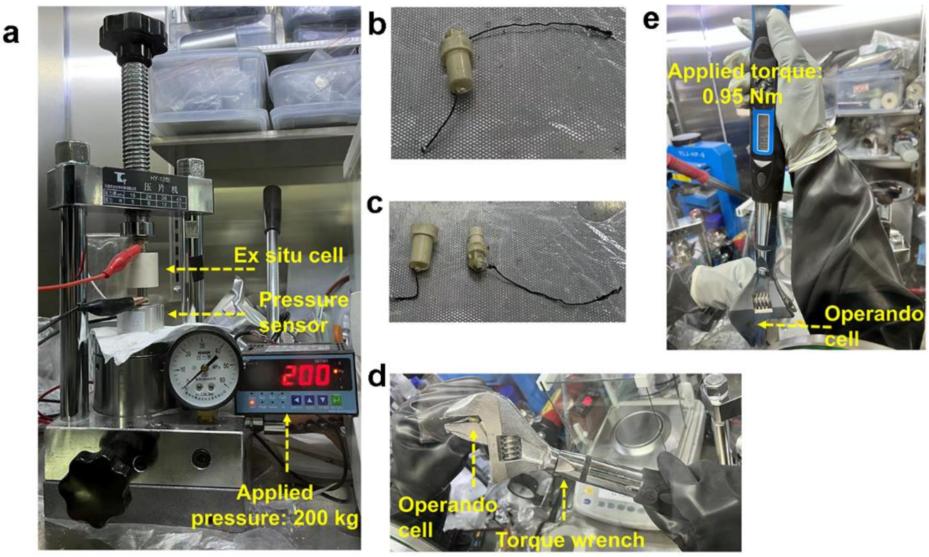

Supplementary Figure 24. Digital images of ex situ/operando cell and corresponding pressurization device. a, ex situ cell and pressure sensor used for pressure calibration, the external pressure is around 25.5 MPa (200 kg). **b, c,** operando cell for NMR in the assemble **(b)** and disassemble **(c)** state. **d,e,** torque wrench used for pressure calibration, the applied torque is around 0.95 Nm, which corresponds to around 907.8 N (23.6 MPa).

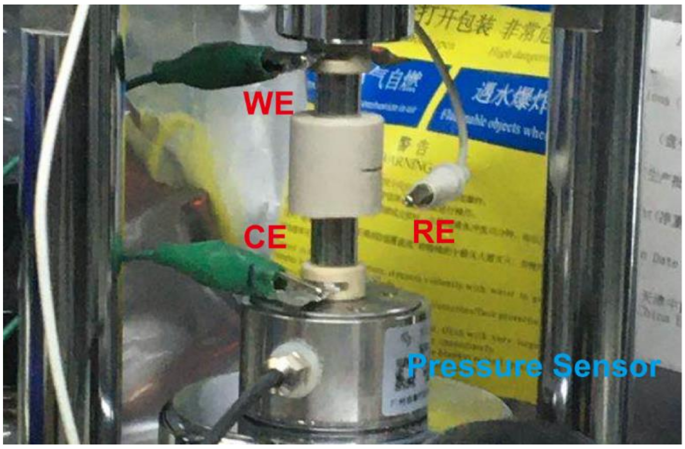

Supplementary Figure 25. Digital image of assembled three-electrode solid state cell.

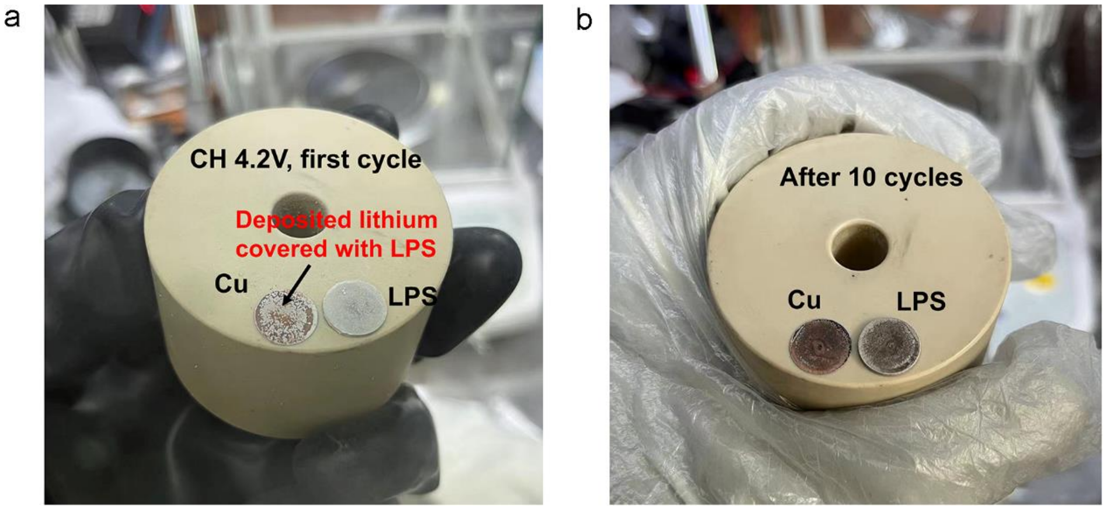

Supplementary Figure 26. Ex situ postmortem photographic picture of the electrode and electrolyte components of the all-solid-state cell. a, cell charging to 4.2 V of the first cycle. **b,** cell after ten cycles. The cells are cycled at a current density of 0.08 mA cm^{-2}, at 30 ± 2 °C, with an applied external pressure of 25 MPa.

Supplementary Table 1. Comparison of Coulombic efficiencies of Li metal cell (with excess lithium metal) and AFBs ($LiCoO_2$ as positive electrode active material). The cells are cycled at a current density of 0.08 mA cm^{-2}, at 30 ± 2 °C, with an applied external pressure of 25 MPa.

	LGPS	LSiPSCl		LPSCl		LPS				
	1st	1st	2nd	1st	2nd	1st	2nd	3rd	4th	5th
Li metal cell	86.1	87.4	96.0	88.9	97.7	83.1	96.9	98.0	98.1	98.5
AFBs	0	82.4	78.0	82.9	82.7	85.5	97.9	91.2	87.2	90.0

Supplementary Table 2. The calculation results of SEI-Li and dead Li of the first cycle of LPS based AFBs without considering corrosion. (discussed in **Supplementary Note** 2)

C_{charge}	$C_{discharge}$	$C_{Li\ depo}$	$C_{dead\ Li}$	$C_{SEI\text{-}Li}$
0.242 mAh	0.198 mAh	0.234 mAh	0.035 mAh (79.5%)	0.009 mAh (20.5%)

Supplementary Table 3. The calculation results of SEI-Li and dead Li of the first cycle of LPS based AFBs with considering corrosion (discussed in **Supplementary Note** 2)

C_{charge}	$C_{discharge}$	C_{corro}	$C_{Li\ depo}$	$C_{dead\ Li}$	C_{SEI-Li}
0.242 mAh	0.198 mAh	0.005 mAh	0.239 mAh	0.036 mAh (81.8%)	0.008 mAh (18.2%)

Supplementary Table 4. The calculation results of SEI-Li and dead Li of the 5th cycle of LPS based AFBs without/with considering corrosion. (discussed in **Supplementary Note** 2)

5th cycle	C_{charge}	$C_{discharge}$	C_{corro}	$C_{Li\ depo}$	$C_{dead\ Li}$	$C_{SEI\text{-}Li}$
Without corrosion	0.079 mAh	0.043 mAh		0.048 mAh	0.005 mAh (13.9%)	0.031 mAh (86.1%)
With corrosion	0.079 mAh	0.043 mAh	0.001 mAh	0.049 mAh	0.005 mAh (13.9%)	0.031 mAh (86.1%)

Supplementary Table 5. The calculation results of SEI-Li and dead Li of the first cycle of LSiPSCl based AFBs without/with considering corrosion. (discussed in **Supplementary Note** 2)

1st cycle	C_{charge}	$C_{discharge}$	C_{corro}	$C_{Li\ depo}$	$C_{dead\ Li}$	$C_{SEI\text{-}Li}$
Without corrosion	0.283 mAh	0.227 mAh		0.250 mAh	0.023 mAh (41.0%)	0.033 mAh (59.0%)
With corrosion	0.283 mAh	0.227 mAh	0.004 mAh	0.254 mAh	0.023 mAh (41.0%)	0.033 mAh (59.0%)

Supplementary Table 6. The calculation results of SEI-Li and dead Li of the 2nd cycle of LSiPSCl based AFBs without/with considering corrosion. (discussed in **Supplementary Note** 2)

2nd cycle	C_{charge}	$C_{discharge}$	C_{corro}	$C_{Li\ depo}$	$C_{dead\ Li}$	$C_{SEI\text{-}Li}$
Without corrosion	0.227 mAh	0.186 mAh		0.197 mAh	0.011 mAh (26.8%)	0.030 mAh (73.2%)
With corrosion	0.227 mAh	0.186 mAh	0.007 mAh	0.205 mAh	0.012 mAh (29.3%)	0.029 mAh (70.7%)

Supplementary Table 7. The calculation results of SEI-Li and dead Li of the 2nd cycle of LPSCl based AFBs without/with considering corrosion. (discussed in **Supplementary Note** 2)

2nd cycle	C_{charge}	$C_{discharge}$	C_{corro}	$C_{Li\ depo}$	$C_{dead\ Li}$	$C_{SEI\text{-}Li}$
Without corrosion	0.197 mAh	0.149 mAh		0.191 mAh	0.042 mAh (87.5%)	0.006 mAh (12.5%)
With corrosion	0.197 mAh	0.149 mAh	0.003 mAh	0.195 mAh	0.043 mAh (89.6%)	0.005 mAh (10.4%)

Supplementary Table 8. The errors between raw and fitted data of EIS spectra in Supplementary Figure 13.

**Li|LGPS|Li**	0h	2h	4h	6h	8h
R_B	5.7%	7.73%	15.3%	22.7%	15.4%
R_{GB}	20.3%	9.05%	38.6%	36.3%	30.9
R_{SEI}	2.72%	2.08%	2.69%	1.86%	2.17%
	10h	12h	14h	16h	18h
R_B	32.5%	27%	25.5%	32.6%	33.7%
R_{GB}	26.7%	26.7%	22.5%	22.4%	27.2%
R_{SEI}	3.48%	3.62%	3.58%	3.42%	3.37%

**Li|LSiPSCl|Li**	0h	2h	4h	6h	8h
R_B	4.2%	0.95%	6.8%	1%	1.2%
R_{GB}	5.7%	4.1%	4.7%	4.1%	1.4%
R_{SEI}	11.6%	4.83%	4.92%	4%	1%
	10h	12h	14h	16h	18h
R_B	7.5%	1.2%	1.4%	1.5%	3.1%
R_{GB}	4.6%	4.6%	6.5%	2.1%	5.2%
R_{SEI}	4.1%	3.78%	4.42%	1%	3.69%

**Li|LPSCl|Li**	0h	2h	4h	6h	8h
R_B	3.1%	4.2%	3.3%	4.5%	5.4%
R_{GB}	13%	12%	16%	14%	8.9%
R_{SEI}	29.7%	15.1%	21.8%	15.2%	10.1%
	10h	12h	14h	16h	18h
R_B	3.1%	3.3%	2.7%	3.8%	3.3%
R_{GB}	15%	8.3%	8.5%	7.9%	7.2%
R_{SEI}	16.4%	11.8%	11.1%	10.6%	9.6%

**Li|LPS|Li**	0h	2h	4h	6h	8h
R_B	0.4%	0.4%	0.9%	0.9%	0.8%
R_{GB}	5.8%	0.3%	4.4%	4.4%	4.4%
R_{SEI}	11.6%	7.4%	6.3%	6%	5.7%
	10h	12h	14h	16h	18h
R_B	0.8%	0.8%	0.7%	0.8%	0.9%
R_{GB}	4.2%	4.2%	4.3%	4%	4.4%
R_{SEI}	5.4%	5.3%	5.4%	5%	5.2%

Supplementary Table 9. The mechanical properties comparison between the four solid state electrolytes. [1,2,3] The density of these SSEs is measured in this work, while the Young's modulus, Shear modulus and Hardness of these SSEs were obtained from literature. The relative density of SSEs pellet is calculated by dividing its actual density by its theoretical density.

	Density/ g cm^{-3}	Theoretical density/ g cm^{-3}	Relative density (porosity)	Young's modulus /GPa	Shear modulus /GPa	Hardness /GPa
LSiPSCl	1.44	–	–	–	–	–
LPSCl	1.49	1.64	90.9% (9.1 %)	21.9	8.1	1.76
LPS	1.72	1.87	92.1% (7.9 %)	22.1	8.1	1.9
LGPS	1.82	1.988	96.6% (3.4 %)	21.7	7.9	–

Supplementary Table 10. The errors between raw and fitted data of EIS spectra in Supplementary Figure 24.

	LGPS	LSiPSCl	LPSCl	LPS
R1	9.36%	5.54%	7.30	9.36%
R2	27.53%	17.67%	47.18%	27.53%

Supplementary Note 1

Calculation the irreversible capacity loss from dead Li and SEI-Li (without considering Li corrosion during discharge)

The charge capacity (C_{charge}) has two contributions: lithium deposition ($C_{Li\ depo}$) and SEI formation ($C_{SEI\text{-}Li}$). The discharge capacity ($C_{discharge}$) represents the dissolution of deposited lithium, but the dissolution process is not complete, thus dead Li ($C_{dead\ Li}$) remains after the dissolution process. Here we neglect the chemical reactions between deposited lithium metal and SSEs during discharge. The following section systematically investigates this issue and proves that the ratio of $C_{dead\ Li}$ and $C_{SEI\text{-}Li}$ change slightly before and after considering corrosion, which will not affect our judgment of the main cause of battery failure.

According to the above definitions, we can obtain the following equations:

$$C_{charge} = C_{Li\ depo} + C_{SEI\text{-}Li} \quad \textbf{(Equation S1)}$$

$$C_{discharge} = C_{Li\ depo} - C_{dead\ Li} \quad \textbf{(Equation S2)}$$

Combining equations S1 and S2 to eliminate $C_{Li\ depo}$, we can obtain:

$$C_{charge} - C_{SEI\text{-}Li} - C_{dead\ Li} = C_{discharge} \quad \textbf{(Equation S3)}$$

Integrating the metallic ^{7}Li NMR signal within the operando NMR spectra, we can quantify the deposited lithium ($S_{Li\ depo}$) and dead lithium ($S_{dead\ Li}$) at the end of charge and discharge, respectively, which is proportional to the $C_{Li\ depo}$ and $C_{dead\ Li}$, respectively. This rational is illustrated in equation S4:

$$C_{Li\ depo} / C_{dead\ Li} = S_{Li\ depo} / S_{dead\ Li} \quad \textbf{(Equation S4)}$$

Combining equations S2 and S4, and cancel $C_{Li\ depo}$, we have :

$$(C_{discharge} + C_{dead\ Li}) / C_{dead\ Li} = S_{Li\ depo} / S_{dead\ Li} \quad \textbf{(Equation S5)}$$

Therefore,

$$C_{dead\ Li} = \frac{S_{dead\ Li}}{S_{deposited} - S_{dead\ Li}} C_{discharge} \quad \textbf{(Equation S6)}$$

According to equations S3

$$C_{SEI\text{-}Li} = (C_{charge} - C_{discharge}) - C_{dead\ Li} \quad \textbf{(Equation S7)}$$

The $\mathbf{C_{charge}}$, $\mathbf{C_{discharge}}$, $\mathbf{S_{Li\ depo}}$, and $\mathbf{S_{dead\ Li}}$ are experimentally observable in operando NMR experiments, thus we can calculate $\mathbf{C_{SEI\text{-}Li}}$ and $\mathbf{C_{dead\ Li}}$, respectively. For example, in LSiPSCl, $\mathbf{C_{charge}}$, $\mathbf{C_{discharge}}$, $\mathbf{S_{Li\ depo}}$, and $\mathbf{S_{dead\ Li}}$ are 0.283 mAh and 0.227 mAh, 1517.22 a.u., 549.46 a.u., respectively for AFBs after first cycle. Therefore, we can calculate that the $\mathbf{C_{SEI\text{-}Li}}$ is 0.033 mAh and $\mathbf{C_{dead\ Li}}$ is 0.023 mAh.

Supplementary Note 2

Calculation the irreversible capacity loss from dead Li and SEI-Li (considering Li corrosion during discharge)

Here, we will evaluate the effect of Li corrosion during discharging on the dead Li and SEI-Li calculations in different SSEs.

(i) For LGPS, the battery cannot discharge due to the chemical reactions between LGPS and deposited Li during charge. Therefore, the corrosion during discharge will not impact the quantification of SEI-Li and dead Li in LGPS.

(ii) For LPS, we will re-calculate the SEI-Li and dead Li when considering corrosion (capacity loss by corrosion during discharge is defined as $\mathbf{C_{corr}}$). **Supplementary Table 2** summaries the calculation results of SEI-Li and dead Li after the first cycle of LPS based AFBs without considering corrosion.

C_{corr} can be estimated from resting experiments (see main text "**Li metal corrosion during calendar aging**"). For the first cycle, the corrosion rate is $(4\% \times C_{Li\ depo})/12$ h, the discharge process of first cycle takes 6.7 h, so $C_{corr} = (4\% \times C_{Li\ depo})/12\ h \times 6.7\ h$. Here, the $C_{Li\ depo}$ is estimated to be the case without considering corrosion (0.234 mAh)[4], therefore, the $C_{corr} = 0.005$ mAh. Then the capacity loss caused by SEI-Li and dead Li can be calculated by combining the following equations, and the results are summarized in **Supplementary Table 3**.

$$\mathbf{C_{charge} = C_{Li\ depo} + C_{SEI\text{-}Li\ charge}} \quad \textbf{(Equation S8)}$$

$$\mathbf{C_{discharge} = C_{Li\ depo} - C_{corr} - C_{dead\ Li}} \quad \textbf{(Equation S9)}$$

$$\mathbf{C_{Li\ depo} / C_{dead\ Li} = S_{Li\ depo} / S_{dead\ Li}} \quad \textbf{(Equation S10)}$$

Combining equations S9 and S10, and cancel $\mathbf{C_{Li\ depo}}$, we have :

$$\mathbf{(C_{discharge} + C_{dead\ Li} + C_{corr}) / C_{dead\ Li} = S_{Li\ depo} / S_{dead\ Li}} \quad \textbf{(Equation S11)}$$

Therefore,

$$\mathbf{C_{dead\ Li} = \frac{S_{dead\ Li}}{S_{deposited} - S_{dead\ Li}} (C_{discharge} + C_{corr})} \quad \textbf{(Equation S12)}$$

Similarly,

$$\mathbf{C_{SEI\text{-}Li} = C_{SEI\text{-}Li\ charge} + C_{corr} = (C_{charge} - C_{discharge}) - C_{dead\ Li}} \quad \textbf{(Equation S13)}$$

After comparing the results in **Supplementary Table 2** and **3**, we can find that the Li corrosion has little effect on the ratio of $C_{dead\ Li}$ and $C_{SEI\text{-}Li}$ in the first cycle. The effects of Li corrosion are also evaluated in 5th cycle, and the results are shown in **Supplementary Table 4**. The ratio of $C_{dead\ Li}$ and $C_{SEI\text{-}Li}$ would not change before and after considering corrosion in the 5th cycle.

(iii) For LSiPSCl, the same calculations were conducted in the 1st and 2nd cycle, as summarized in **Supplementary Table 5 and 6**. Similarly, Li corrosion during discharge also has weak effect on the ratio of $C_{dead\ Li}$ and $C_{SEI\text{-}Li}$.

(iv) For LPSCl, almost no Li corrosion occurred in the first cycle. The Li corrosion in the 2nd cycle is summarized in **Supplementary Table 7**. Similarly, it seems that Li corrosion has little effect on the ratio of $C_{dead\ Li}$ and $C_{SEI\text{-}Li}$.

In conclusion, the ratio of $C_{dead\ Li}$ and $C_{SEI\text{-}Li}$ change slightly before and after considering corrosion. From a qualitative point of view, this slight change will not affect our judgment of the main cause of battery failure, i.e., our conclusions are reliable even without considering corrosion. Of course, we can further differentiate the total SEI after considering corrosion. It can be find that in some cases, the corrosion induced SEI would account for around half of the total SEI, indicating the important role of corrosion in forming SEI.

Supplementary Note 3
Interphase between the solid state electrolytes and lithium metal

The stability of the SEI also has a great influence on the electrochemical performance of the battery. The formation of SEI not only consumes active lithium but also increases the impedance of the batteries. As a consequence, the interfacial stability of these four SSEs was compared by monitoring their impedance increment of the assembled Li|SSEs|Li cells during resting. It is obvious from **Supplementary Figure 12** that the impedance of the Li|LGPS|Li cell shows an increasing trend during the resting process, while the impedance of the other three systems mainly increases after 2 h of resting, after which the impedance grows slowly. By fitting the impedance spectra (**Supplementary Figure 13a**), we could obtain bulk resistance of SSEs (R_B), grain boundary resistance of SSEs (R_{GB}), and resistance of SEI (R_{SEI}). (**Supplementary Figure 13b, 13c 13d, and 13e**). It can be seen that R_B remains constant during rest, but the R_{GB} and R_{SEI} increases during rest. More specifically, the R_{SEI} and R_{GB} of cells with LGPS exhibits a continuous increase during rest, while the R_{SEI} and R_{GB} of battery with other three SSEs mainly increase at the start of rest. The R_{SEI} is determined by the thickness and composition (affect transport of Li^+) of SEI, whose evolution indicates that the interfacial deterioration of the Li|LGPS|Li battery is continuous, whereas in the other three systems the interface is relatively stable once it is formed. The evolution of R_{GB} (**Supplementary Figure 13d**) is similar with that of R_{SEI} (**Supplementary Figure 13e**). Therefore, we consider that Li metal infiltrates the grain boundary of SSEs sub-surface due to the initial applied pressure during cell fabrication, which is verified by the previous report[5]. The Li metal located at the grain boundary would react with adjacent SSEs to form SEI, corresponding to the increase of R_{GB}.

The composition of the SEI has a great influence on the performance of both LIBs and liquid/solid-state LMBs, so an in-depth understanding of the composition of SEI is of great importance.[6] For the LGPS sample, the SEI compositions have been identified to include Li_2S, Li_3P and Li-Ge alloy.[7] While for LPS and LPSCl samples, previous studies showed that the SEI composition contained Li_2S, Li_3P, and LiCl.[8] So far, the SEI composition of LSiPSCl is still unknown. Therefore, a Li|LSiPSCl|Li symmetrical cell was assembled, which showed a gradual increase in polarization voltage during cycling (**Supplementary Figure 14**). The cycled lithium metal was carefully stripped off and the pellet surface was characterized by X-ray photoelectron spectroscopy (XPS) The XPS results are shown in **Supplementary Figure 15**. The Cl 2*p* spectra show a negligible peak shift before and after cycling. The signal of Li_2S is observed from the Li 1*s* and S 2*p* spectra. The little sulfate peak in the S 2*p* spectrum at 167 eV may be due to the reaction of the sample with traces of residual oxygen during sample transfer. In the Si 2*p* spectrum, the pristine LSiPSCl shows a peak at 100.75 eV, corresponding to the Si-S bond of SiS_4 tetrahedron. The broad peak at 102.2 eV can be assigned to the Si-Cl bond of $SiCl_4$ tetrahedron. After cycling, the peak at 100.75 eV shifts to a lower energy at 100.6 eV. This small change is often thought to be a change in the local chemical environment of Si. We speculate that Si is bounded with less electronegative

atoms after cycling, thus increasing the electron density on silicon. We proposed that SiS_4^{4-} is reduced to $Si_2S_6^{6-}$, meanwhile Li_2S is generated, in which some S-Si-S bonds are converted to S-Si-Si-S bonds, the chemical equation can be refered in **Supplementary Figure 15f**. This mechanism is same with the Si-contained glassy sulfide SSEs reported previously.[9] A similar peak shift to lower energy is observed in the P 2*p* spectra, which we assigned it as PS_4^{3-} converting into $P_2S_6^{4-}$.

Supplementary Note 4
Low chemical shift of deposited lithium in SSBs

The chemical shift of Li metal in different SSEs gradually shifts from ~240 ppm to ~ 230 ppm during charge of first cycle (**Figure 3b** and **Supplementary Figure 10**). And it is noted that such a chemical shift is lower than that of in liquid electrolyte based LMBs (240 – 275 ppm). Several factors could affect the chemical shift of Li metal, including the orientation of deposited Li with respect to external magnetic field, the morphology of Li metal, and bulk magnetic susceptibility (BMS) of cathode.

Firstly, the orientation of deposited lithium with respect to external magnetic field in solid state battery is shown in **Supplementary Figure 16a,** which is fixed during battery operation.

Secondly, the effect of morphology of deposited Li metal is discussed with the following experiments. We first assemble a Cu||LFP cell using a 1M LiPF6 in EC:EMC (3:7 w/w + 2 wt.% VC additive) non-aqueous electrolyte solution and monitor the lithium deposition process with operando NMR. The deposited lithium shows a porous morphology (**Supplementary Figure 16b**) and its chemical shift is at around 250 ppm (**Supplementary Figure 16c**). The morphology of lithium metal in liquid electrolyte-based battery is less damaged when the battery is disassembled. So the deposited lithium was retrieved and used to re-assemble an operando solid state lithium metal battery with pristine LCO (cathode) and LPS (solid state electrolyte). The operando cell is free of external pressure so as to maintain the porous morphology of lithium metal. The orientation of deposited lithium with respect to external magnetic field in solid state battery and in liquid electrolyte based battery is same, as schematic in **Supplementary Figure 16a**. The re-assembled battery was also characterized by operando NMR and the chemical shift of lithium metal shows almost no change (**Supplementary Figure 16c**), indicating that pristine LCO and LPS will not influence the chemical shift of lithium metal. The lithium metal was retrieved again and pressed at 50 MPa, exhibiting a relatively compact and flat morphology **(Supplementary Figure 16d**). The pressed lithium metal was reassembled an operando cell with LPS and LCO for NMR testing. The chemical shift of pressed lithium metal shifts to lower chemical shift around 236 ppm, which is lower than that typically observed, thus proving that the compact morphology of lithium metal leads to a low chemical shift. The morphology of lithium metal in SSBs is even more compact than that of pressed lithium metal. Therefore, we consider that the unique compact morphology of Li metal is probably one of the reasons.

Finally, we also investigate the effect of LCO to chemical shift of Li metal. The pristine LCO is diamagnetic, it has negligible effect to the chemical shift of underlying Li metal, as demonstrated by the experiments in **Supplementary Figure 16**. We further explore the effect of charged LCO (paramagnetic) to the chemical shift of Li metal. The AFBs was firstly charged to 4.2 V, then the cathode (corresponding to formula of $Li_{0.5}CoO_2$)

was collected and was used to re-prepare a new pellet with LPS. A lithium metal with thickness of 370 μm was used to paired with this new pellet for NMR testing. The results are shown in **Supplementary Figure 17**, we can find that the signal of Li metal shifts to lower chemical shift when $Li_{0.5}CoO_2$ is used, which is the same as the signal shift to a lower chemical shift during the 1st charging process observed in the operando experiments (**Figure 3b, Supplementary Figure 10**). So the paramagnetic cathode could also affect the chemical shift of Li metal.

In summary, we tentatively ascribe the low ^{7}Li chemical shift of Li metal to the synergistic effects of delithiated cathode and morphologies of deposited Li.

Supplementary Note 5
Calculation processes for converting torque into applied force

The applied force can be calculated as:
(https://www.engineeringtoolbox.com/screw-jack-d_1308.html)

$$\mathrm{F} = \frac{T}{r} \times \frac{(2\pi r - \mu p)}{(2\pi\mu r + p)} \quad \text{(Equation S3)}$$

T: torque, in this work, is around 0.95 Nm
r: the radius of screw, in this work, is 3.5 mm
p: screw pitch, in this work, is 1 mm
μ: the friction coefficient, the material of screw is poly(ether ether ketone) cylinder (PEEK), whose friction coefficient is 0.25 (https://www.curbellplastics.com/Research-Solutions/Materials/PEEK).

The calculated force is around 907.8 N (23.6 MPa).

Supplementary Note 6
Sample preparation of SEM

In our cases, the copper is easily peeled off from the SSEs, and the deposited lithium remains on copper but is partially covered with SSEs (**Supplementary Figure 26a**). Therefore, we think that the morphology of deposited lithium is relatively less damaged in this process, but the SEI on deposited lithium might be totally destroyed. When we observe the morphology of deposited lithium by SEM, we need to search carefully for the deposited lithium without covering SSEs. After multiple cycles, the SSEs pellet is nearly intact after peeling off the copper (**Supplementary Figure 26b**), but we need to break the pellet to find the lithium dendrite/dead lithium inside the pellet.

Reference:

1 Deng, Z., Wang, Z., Chu, I.-H., Luo, J. & Ong, S. P. Elastic Properties of Alkali Superionic Conductor Electrolytes from First Principles Calculations. *Journal of The Electrochemical Society* **163**, A67-A74, doi:10.1149/2.0061602jes (2015).

2 Kim, K. J., Balaish, M., Wadaguchi, M., Kong, L. & Rupp, J. L. M. Solid-State Li–Metal Batteries: Challenges and Horizons of Oxide and Sulfide Solid Electrolytes and Their Interfaces. *Advanced Energy Materials* **11**, 2002689, doi:https://doi.org/10.1002/aenm.202002689 (2020).

3 Papakyriakou, M. *et al.* Mechanical behavior of inorganic lithium-conducting solid electrolytes. *Journal of Power Sources* **516**, 230672, doi:https://doi.org/10.1016/j.jpowsour.2021.230672 (2021).

4 Gunnarsdóttir, A. B., Amanchukwu, C. V., Menkin, S. & Grey, C. P. Noninvasive In Situ NMR Study of “Dead Lithium” Formation and Lithium Corrosion in Full-Cell Lithium Metal Batteries. *Journal of the American Chemical Society* **142**, 20814-20827, doi:10.1021/jacs.0c10258 (2020).

5 Doux, J.-M. *et al.* Stack Pressure Considerations for Room-Temperature All-Solid-State Lithium Metal Batteries. *Advanced Energy Materials* **10**, 1903253, doi:10.1002/aenm.201903253 (2019).

6 Winter, M. The Solid Electrolyte Interphase – The Most Important and the Least Understood Solid Electrolyte in Rechargeable Li Batteries. *Zeitschrift für Physikalische Chemie* **223**, 1395-1406, doi:doi:10.1524/zpch.2009.6086 (2009).

7 Wenzel, S. *et al.* Direct Observation of the Interfacial Instability of the Fast Ionic Conductor Li10GeP2S12 at the Lithium Metal Anode. *Chemistry of Materials* **28**, 2400-2407, doi:10.1021/acs.chemmater.6b00610 (2016).

8 Wenzel, S., Sedlmaier, S. J., Dietrich, C., Zeier, W. G. & Janek, J. Interfacial reactivity and interphase growth of argyrodite solid electrolytes at lithium metal electrodes. *Solid State Ionics* **318**, 102-112, doi:https://doi.org/10.1016/j.ssi.2017.07.005 (2018).

9 Takahara, H. *et al.* Application of Lithium Metal Electrodes to All-Solid-State Lithium Secondary Batteries Using Li3PO4-Li2S-SiS2 Glass. *Journal of The Electrochemical Society* **151**, A1309, doi:10.1149/1.1773712 (2004).
